# An approach to estimating the environmental burden of cancer from known and probable carcinogens: application to Ontario, Canada

**DOI:** 10.1186/s12889-020-08771-w

**Published:** 2020-06-26

**Authors:** Susan Lavinia Greco, Elaina MacIntyre, Stephanie Young, Hunter Warden, Christopher Drudge, JinHee Kim, Elisa Candido, Paul Demers, Ray Copes

**Affiliations:** 1 Public Health Ontario, Toronto, Ontario Canada; 2 Dalla Lana School of Public Health, University of Toronto, Toronto, Ontario Canada; 3 Cancer Care Ontario, Toronto, Ontario Canada; 4 Occupational Cancer Research Centre, Cancer Care Ontario, Toronto, Ontario Canada; 5 Institute for Clinical Evaluative Sciences, Toronto, Ontario Canada; 6 School of Population and Public Health, University of British Columbia, Vancouver, British Columbia Canada

**Keywords:** Carcinogen, Environment, Cancer, Burden of disease, Burden of cancer, Probabilistic modeling, Risk assessment, Population attributable fraction, Pollutant

## Abstract

**Background:**

Quantifying the potential cancer cases associated with environmental carcinogen exposure can help inform efforts to improve population health. This study developed an approach to estimate the environmental burden of cancer and applied it to Ontario, Canada. The purpose was to identify environmental carcinogens with the greatest impact on cancer burden to support evidence-based decision making.

**Methods:**

We conducted a probabilistic assessment of the environmental burden of cancer in Ontario. We selected 23 carcinogens that we defined as “environmental” (e.g., pollutants) and were relevant to the province, based on select classifications provided by the International Agency for Research on Cancer. We evaluated population exposure to the carcinogens through inhalation of indoor/outdoor air; ingestion of food, water, and dust; and exposure to radiation. We obtained or calculated concentration-response functions relating carcinogen exposure and the risk of developing cancer. Using both human health risk assessment and population attributable fraction models in a Monte Carlo simulation, we estimated the annual cancer cases associated with each environmental carcinogen, reporting the simulation summary (e.g., mean and percentiles).

**Results:**

We estimated between 3540 and 6510 annual cancer cases attributable to exposure to 23 environmental carcinogens in Ontario. Three carcinogens were responsible for over 90% of the environmental burden of cancer: solar ultraviolet (UV) radiation, radon in homes, and fine particulate matter (PM_2.5_) in outdoor air. Eight other carcinogens had an estimated mean burden of at least 10 annual cancer cases: acrylamide, arsenic, asbestos, chromium, diesel engine exhaust particulate matter, dioxins, formaldehyde, and second-hand smoke. The remaining 12 carcinogens had an estimated mean burden of less than 10 annual cancer cases in Ontario.

**Conclusions:**

We found the environmental burden of cancer in Ontario to fall between previously estimated burdens of alcohol and tobacco use. These results allow for a comparative assessment across carcinogens and offer insights into strategies to reduce the environmental burden of cancer. Our analysis could be adopted by other jurisdictions and repeated in the future for Ontario to track progress in reducing cancer burden, assess newly classified environmental carcinogens, and identify top burden contributors.

## Background

Cancer is one of the leading causes of death worldwide [1] and the leading cause of mortality in Ontario, Canada [2]. A 2018 report by Cancer Care Ontario found that Ontarians have a 1 in 2 chance of being diagnosed with cancer in their lifetime [2]. Furthermore, cancer prevalence in Canada is expected to increase substantially over the next decade due to the aging population [3]. A substantial proportion of cancer cases are attributable to avoidable risk factors [4, 5], making prevention an important strategy for reducing the burden of cancer. Carcinogens in the environment comprise one potentially avoidable risk factor. Understanding the comparative cancer burden from carcinogenic exposures may help decision-makers to prioritize carcinogens for additional policy interventions to reduce exposures and risks. There have been multiple recent efforts to estimate the cancer burden associated with lifestyle, behavioral, and environmental factors in the United States [6], the United Kingdom [7, 8], Australia [9], and Alberta, Canada [5]. However, these studies restricted their examination of environmental carcinogens to air pollution, second-hand smoke, and radiation sources (e.g., radon and solar UV). As such, they did not examine many carcinogens that exist in air, water, and soil such as persistent organic pollutants (e.g., dioxins) or metals (e.g., arsenic). On the other hand, there have been broad environmental cancer assessments examining pollutants, though these have not used a systematic process to select the environmental carcinogens. For example, Hänninen and Knol examined the environmental burden of cancer from dioxin and benzene (in addition to air pollution, radon, and second-hand smoke) in Europe, using a group of environmental health experts to select the carcinogens based on public health impact, individual risk, and political/public concern [10].Hänninen and Knol applied one method to all carcinogens, regardless of the type of toxicity information that was available. Woodruff et al. estimated excess cancer risk for hazardous air pollutants in the United States, such as formaldehyde, benzene, chromium, and 1,3-butadiene [11].

In this study, we wished to examine the relative burden of incident cancers from carcinogens in the environment with potential for community exposures. We used a systematic process to select the carcinogens. We employed established methods to examine the burden of cancer, using the method that was most appropriate to the available toxicity information. The objective of this study was to compare the cancer burden across environmental carcinogens and exposure pathways to better understand the relative public health impact of each.

## Methods

This study aimed to evaluate the cancer burden associated with exposure to select environmental carcinogens with potential for community exposure in Ontario, Canada. We identified carcinogen concentrations for multiple routes of exposure and relevant toxicity information (concentration-response relationships). Based on the toxicity information available for the carcinogen, we employed a suitable method to estimate the cancer burden in a probabilistic analysis.

### Carcinogen selection

We followed the general approach of Kauppinen et al. (2000) [12] and Setton et al. (2013) [13] to systematically select environmental carcinogens starting with reviews of carcinogenic agents available from the International Agency for Research on Cancer (IARC) [13]. At the time of our study, a total of 188 agents had been identified by IARC as “carcinogenic to humans” (Group 1) or “probably carcinogenic to humans” (Group 2A). We identified 52 of the 188 agents as having potential for exposure in the community environment, whether through air, food, water, dust, or other pathways. We excluded 136 agents with low likelihood of community exposure. Namely, agents we classified as: occupational (e.g., coal tar pitch; *n* = 57), pharmacological (e.g., tamoxifen; *n* = 38), microbiological (e.g., aflatoxin; *n* = 15), nutritional (e.g., betel quid; *n* = 11), hormonal (e.g., estrogen therapy; *n* = 7), radionuclide (e.g., plutonium, n = 7), or behavioral (i.e., direct smoking or use of tanning beds; *n* = 2). The full classification details are presented in Additional file 1 and elsewhere [14].

**Fig. 1:**
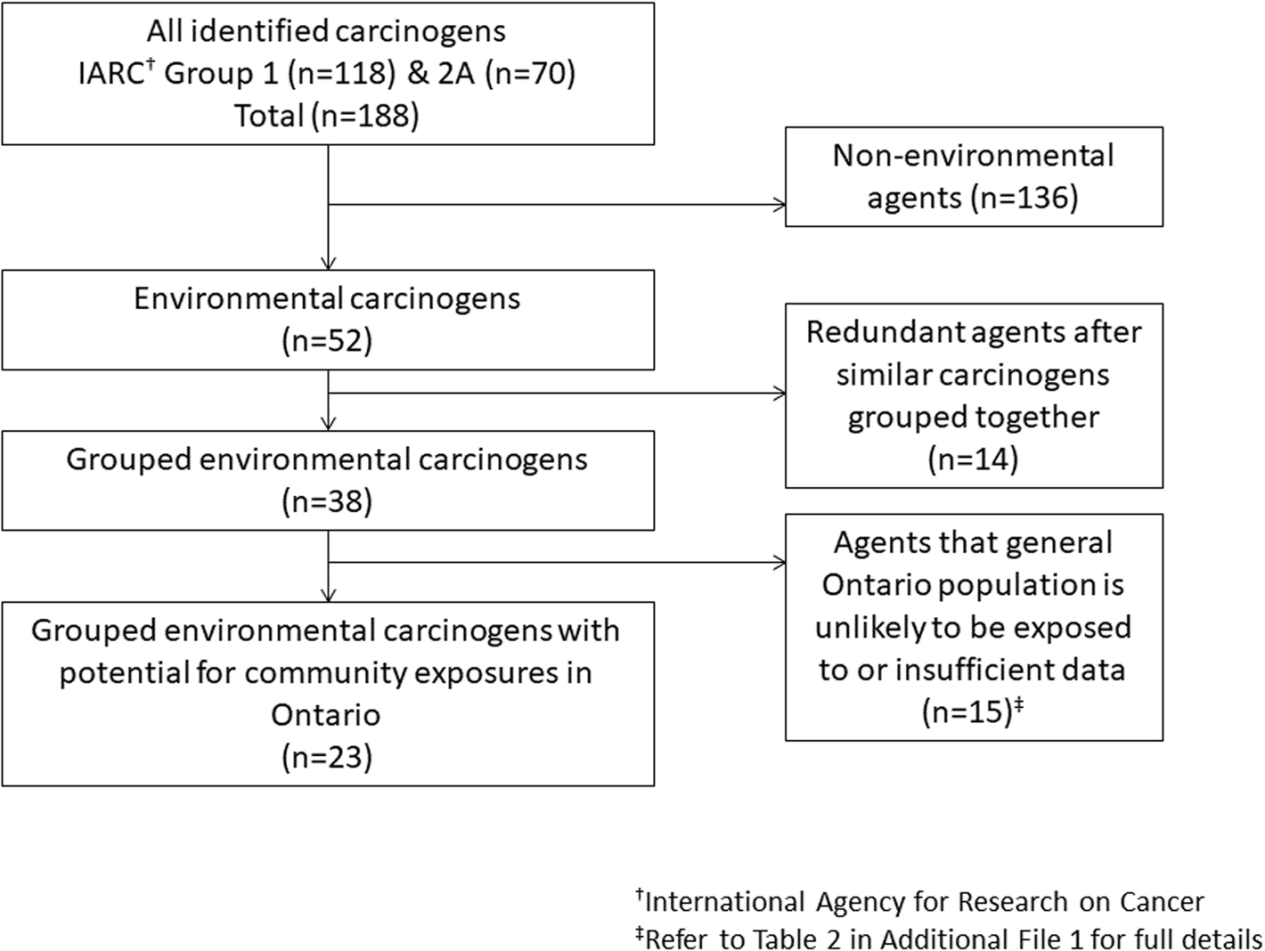
Exclusion flowchart for selecting environmental carcinogens

Of the 52 environmental agents, we grouped related carcinogens together (e.g., different forms of polycyclic aromatic hydrocarbons), reducing the number of agents to 38. We then excluded 14 agents after determining, upon further evaluation, that the general population in Ontario would not be exposed to them (e.g., indoor coal emissions) and one agent due to data insufficiency (i.e., silica dust). In the end, 23 carcinogens met our inclusion criteria (see Fig. 1 for flowchart outlining the environmental carcinogen selection; Tables 1 and 2 list the 23 carcinogens included in this study).

**Table 1:** Potency values (by agency)

Carcinogen	Oral Slope Factor (per mg/kg-day)			Inhalation Unit Risk (per μg/m^**3**^)		
	Health Canada	US EPA	CalEPA	Health Canada	US EPA	CalEPA
**1,2-Dichloropropane**			3.6E-02			1.0E-05
**1,3-Butadiene**			6.0E-01		3.0E-05	1.7E-04
**2,3,7,8-Tetrachlorodibenzo-para-dioxin (TCDD)**			1.3E+ 05			3.8E+ 01
**Acrylamide**		5.0E-01	4.5E+ 00		1.0E-04	1.3E-03
**Alpha-Chlorinated toluenes**		1.7E-01	1.7E-01			4.9E-05
**Arsenic**	1.8E+ 00	1.5E+ 00	9.5E+ 00	6.4E-03	4.3E-03	3.3E-03
**Asbestos**^**a**^					2.3E-01	1.9E+ 00
**Benzene**^**b**^	8.3E-02	5.5E-02	1.0E-01	3.3E-06	7.8E-06	2.9E-05
**Cadmium**^**c**^				9.8E-03	1.8E-03	4.2E-03
**Chromium (VI)**			5.0E-01	7.6E-02	1.2E-02	1.5E-01
**Dichloromethane (methylene chloride)**	7.9E-05	2.0E-03	1.4E-02	2.3E-08	1.0E-08	1.0E-06
**Diesel particulate matter**						3.0E-04
**Formaldehyde**					1.3E-05	6.0E-06
**Nickel**						2.6E-04
**Polycyclic Aromatic Hydrocarbons (PAHs)**	2.3E+ 00	7.3E+ 00	2.9E+ 00	3.1E-05		1.1E-03
**Polychlorinated biphenyls (PCBs)**		2.0E+ 00	2.0E+ 00		1.0E-04	5.7E-04
**Tetrachloroethylene (PCE)**		2.1E-03	5.4E-01		2.6E-07	5.9E-06
**Trichloroethylene (TCE)**	8.1E-04	4.6E-02	5.9E-03	6.1E-07	4.1E-06	2.0E-06
**Vinyl chloride (chloroethene)**^**d**^	2.6E-01	1.5E+ 00	2.7E-01		8.8E-06	7.8E-05

^a^The units for the asbestos IUR are per fibres/mL

^b^Where one agency presented a range for the slope factor, the high range from that agency was used

^c^While CalEPA presented an OSF for cadmium we did not employ it

^d^The “from birth” value was selected from US EPA IRIS

**Table 2:** Population attributable fractions for selected environmental carcinogens in Ontario, Canada

Carcinogen	Cancer site evaluated	Mean PAF % (5th, 95th PCT)^f^
PM_2.5_^a^ DPM^a^	Lung	5.8 (3.0, 9.4)
Radon^b^	Lung	13.6 (11.2, 16.0)
Second-hand smoke^c^	Lung	0.6 (0.1, 1.3)
Solar UV radiation^d^	Skin^e^	79.7 (65.6, 93.8)

*Abbreviations*: *5th* 95th PCT, 5th and 95th percentile estimates, *DPM* diesel particulate matter, *NA* not available, *PAF* population attributable fraction, *PM*_*2.5*_ fine particulate matter, *UV* ultraviolet

^a^DPM was treated as a sub-exposure of PM_2.5_

^b^The radon PAF accounted for the different risks associated with ever-smokers and never-smokers

^c^Relative risks were for never-smokers exposed to second-hand smoke in their homes; PAF accounted for the different risks associated with females and males

^d^Two methods were used to estimate attributable melanoma cases and an uniform distribution was fit using the estimates as the lower and upper bounds

^e^Only includes melanoma skin cancer

^f^See Additional file 4 for more information

### Exposure concentration

We tried to characterize environmental exposures from five routes: indoor air, outdoor air, food, drinking water, and indoor dust. We fit log-normal, normal, or discrete probability distributions to the carcinogen concentration data for each route of exposure that had sufficient data. We selected environmental concentration data that were representative of exposures for the Ontario population. Exposure data sources included government regulatory monitoring data, surveillance programs, and baseline studies. For individual concentrations that were below the limit of detection (LOD), we replaced the non-detects with the LOD/√2. The mean, 5th and 95th percentile concentration estimates and the sources that the estimates were based on are summarized in Table 3. Additional concentration details are presented in Additional file 2 and elsewhere [14].

**Table 3:** Mean, 5th and 95th percentile exposure concentrations for environmental carcinogens by route of exposure

Environmental carcinogen	Route of exposure	Mean (5th, 95th PCT)	Units	Reference
1,2-Dichloropropane	Outdoor air	0.017 (0.0069, 0.032)	μg/m^3^	NAPS [14]
	Indoor air	0.011 (0.0046, 0.022)	μg/m^3^	Zhu et al. [15]
	Drinking water	0.050 (NA)	μg/L	DWSP [16]
1,3-Butadiene	Outdoor air	0.047 (0.0021, 0.17)	μg/m^3^	NAPS [14]
	Indoor air	0.15 (0.030, 0.40)	μg/m^3^	Health Canada [17]
Acrylamide	Food	0.28 (0.16, 0.60)	μg/kg · day	AMP [18]
Alpha-chlorinated toluenes	Outdoor air	0.012 (0.0027, 0.031)	μg/m^3^	NAPS [14]
	Indoor air	0.016 (0.014, 0.018)	μg/m^3^	Health Canada [17]
Arsenic	Outdoor air	0.69 (0.10, 2.0)	ng/m^3^	NAPS [14]
	Indoor air	0.18 (0.025, 0.55)	ng/m^3^	Bari et al. [19]
	Drinking water	0.47 (0.15, 1.06)	μg/L	DWSP [16]
	Food	0.57 (0.37, 0.94)	μg/kg · day	CTDS [20]
	Dust	18 (2.1, 38)	μg/g	CHDS [21]
Asbestos	Outdoor air	0.00021 (0.000017, 0.00050)	fibers/mL	Lee et al. [22]
	Indoor air	0.00026 (0.000023, 0.00062)	fibers/mL	Lee et al. [22]
Benzene	Outdoor air	0.51 (0.12, 1.3)	μg/m^3^	NAPS [14]
	Indoor air	1.9 (0.18, 6.1)	μg/m^3^	Zhu et al. [15]
	Drinking water	0.050 (NA)	μg/L	DWSP [16]
Cadmium	Outdoor air	0.11 (0.022, 0.31)	ng/m^3^	NAPS [14]
	Indoor air	0.032 (0.0071, 0.084)	ng/m^3^	Bari et al. [19]
Chromium (VI)	Outdoor air	0.40 (0.10, 1.0)	ng/m^3^	NAPS [14]
	Indoor air	1.0 (0.11, 3.3)	ng/m^3^	Bari et al. [19]
	Drinking water	0.29 (0.053, 0.78)	μg/L	DWSP [16]
	Dust	147 (19, 310)	μg/g	CHDS [21]
Dichloromethane	Outdoor air	0.37 (0.13, 0.81)	μg/m^3^	NAPS [14]
	Indoor air	1.9 (0.38, 5.0)	μg/m^3^	Health Canada [17]
	Drinking water	0.20 (NA)	μg/L	DWSP [16]
DPM	Outdoor air	0.98 (0.17, 2.8)	μg/m^3^	CARB [23]
Formaldehyde	Outdoor air	1.9 (0.33, 5.5)	μg/m^3^	NAPS [14]
	Indoor air	30 (12, 61)	μg/m^3^	Héroux et al. [24]
Nickel	Outdoor air	0.51 (0.082, 1.5)	ng/m^3^	NAPS [14]
	Indoor air	1.0 (0.036, 3.9)	ng/m^3^	Bari et al. [19]
PAHs	Outdoor air	0.099 (0.0038, 0.37)	ng/m^3^	NAPS [14]
	Indoor air	0.21 (0.013, 0.76)	ng/m^3^	Li et al. [25]
	Drinking water	1.0 (NA)	ng/L	DWSP [16]
	Food	55 (30, 90)	ng/day	Kazerouni et al. [26]
	Dust	1.8 (0.15, 6.3)	μg/g	Maertens et al. [27]
PCBs	Outdoor air	0.0031 (0.00039, 0.0095)	pg of TEQ/m^3^	NAPS [14]
	Indoor air	0.19 (0.046, 0.47)	pg of TEQ/m^3^	Harrad et al. [28]
	Food	0.081 (0.049, 0.22)	pg of TEQ/kg · day	CTDS [20]
	Dust	0.0096 (0.0017, 0.019)	ng of TEQ/g	Harrad et al. [28]
PM_2.5_	Outdoor air	5.8 (3.8, 8.3)	μg/m^3^	AQO [29]
TCDD (dioxins)	Outdoor air	0.014 (0.0028, 0.040)	pg of TEQ/m^3^	NAPS [14]
	Food	0.80 (0.44, 2.4)	pg of TEQ/kg · day	CTDS [20]
Tetrachloroethylene (PCE)	Outdoor air	0.088 (0.017, 0.24)	μg/m^3^	NAPS [14]
	Indoor air	0.71 (0.037, 2.6)	μg/m^3^	Zhu et al. [15]
	Drinking water	0.053 (0.033, 0.080)	μg/L	DWSP [16]
Trichloroethylene (TCE)	Outdoor air	0.038 (0.0037, 0.13)	μg/m^3^	NAPS [14]
	Indoor air	0.065 (0.0079, 0.20)	μg/m^3^	Zhu et al. [15]
	Drinking water	0.053 (0.036, 0.075)	μg/L	DWSP [16]
Vinyl chloride	Outdoor air	0.0027 (0.00074, 0.0063)	μg/m^3^	NAPS [14]
	Indoor air	0.025 (0.020, 0.030)	μg/m^3^	Health Canada [17]
	Drinking water	0.050 (NA)	μg/L	DWSP [16]

*Abbreviations*: *5th* 95th PCT, 5th and 95th percentiles, *AMP* Acrylamide Monitoring Program, *AQO* Air Quality Ontario, *CARB* California Air Resources Board, *CHDS* Canadian House Dust Study, *CTDS* Canadian Total Diet Study, *DPM* diesel engine exhaust particulate matter, *DWSP* Drinking Water Surveillance Program, *NA* not available, *NAPS* National Air Pollution Surveillance Program, *PAHs* polycyclic aromatic hydrocarbons, *PCBs* polychlorinated biphenyls, *PM*_*2.5*_ fine particulate matter, *TCDD* 2,3,7,8-tetrachlorodibenzo-para-dioxin, *TEQ* toxic equivalency factor

### Probabilistic approach

We used a probabilistic approach to estimate the annual number of cancer cases attributable to each environmental carcinogen. The probabilistic approach incorporated known variability and uncertainty in the inputs, characterizing them as distributions rather than point estimates, following Hanninen et al. [10], Gakidou et al. [15], and P-U et al. [16]. We performed Monte Carlo simulations using the @RISK add-in for Excel (version 7.5.2, Pallisade Corporation, 2016) to estimate the annual cancer burden associated with each carcinogen. We reported the mean and 90% prediction interval (90% PI) for each burden estimate. The number of iterations was 10,000 and the sampling type was Latin Hypercube [17]. We used one of two established models to estimate the annual cancer burden based on the nature of the concentration-response functions that were available for each carcinogen: risk assessment and population attributable fraction.

### Risk assessment model

Human health risk assessment frameworks have been widely employed by agencies such as the United States Environmental Protection Agency [18], Health Canada [19], and the World Health Organization [20] to estimate excess cancer risk from lifetime exposure to carcinogens. We used the risk assessment model for 19 of the 23 selected carcinogens (see Table 1). We used exposures for the most recent year available and assumed these exposures would continue over a lifetime.

Conceptually, lifetime attributable cancer cases were estimated by the following formula:

$$\mathrm{Attributable}\ \mathrm{Cancers}=\mathrm{Average}\ \mathrm{Lifetime}\ \mathrm{Exposure}\bullet \mathrm{Potency}\bullet \mathrm{Population}\ \mathrm{Exposed}$$

This was divided by 80 years to give an annual attributable number of future cancers. The full details, inputs, and equations are described in Additional file 3 and elsewhere [14]. Briefly, the annual cancer burden estimate for each carcinogen was the sum of the estimates for each potential route of exposure where there were available input data for more than one route of exposure. For the annual cancer estimates for indoor and outdoor air, the potency was the inhalation unit risk (units: inverse μg/m^3^), and we assumed Ontario residents spend 96% of their time, on average, indoors [21]. For the annual cancer estimates for drinking water, food, and indoor dust, the potency was the oral slope factor (units: inverse mg/kg-d) and we applied appropriate exposure factor distributions for drinking water ingestion rates (L/d), dust ingestion rates (mg/d), and bodyweights (kg) [21, 22]. The population of Ontario under the age of 80 was approximately 12.7 million in the year 2011 [23].

The oral slope factors and inhalation unit risks were typically derived from models applied to animal studies or occupational studies where animal subjects or human participants were exposed to high levels of the carcinogen. As defined by the agencies, they represent an upper bound on cancer risk rather than a central estimate. We obtained oral slope factors and inhalation unit risks from Health Canada [24], the United States Environmental Protection Agency [25], and the California Environmental Protection Agency [26]. If more than one oral slope factor or inhalation unit risk was available for a carcinogen, the potency estimate was modeled as a discrete uniform probability distribution. All potency values for each carcinogen are listed in Table 1.

### Population attributable fraction model

It was not possible to apply a risk assessment model to all carcinogens based on the nature of the concentration-response data available. As such, we based our approach on previous literature and employed a population attributable fraction (PAF) model [5, 7–10, 15, 16].

We estimated current annual attributable cancer cases associated with 5 of the 23 selected carcinogens (listed in Table 2) using the following general formula:

$$\mathrm{Annual}\ \mathrm{Attributable}\ \mathrm{Cancer}\mathrm{s}=\mathrm{Population}\ \mathrm{Attributable}\ \mathrm{Fraction}\bullet \mathrm{Annual}\ \mathrm{Cancer}\ \mathrm{Incidence}$$

Details of the PAF models we developed for each carcinogen can be found in Additional file 4 and elsewhere [14] and the values are summarized in Table 2. Briefly, for fine particulate matter (PM_2.5_), the PAF was a function of provincial annual average PM_2.5_ exposure [30] and a relative risk from a meta-analysis conducted by Hamra et al. [27] that demonstrated a relationship between PM_2.5_ exposure and lung cancer. Diesel engine exhaust particulate matter (DPM) was treated as a subset of PM_2.5_. The same relative risk was used for DPM as PM_2.5_ and the environmental DPM concentration was derived from a county-level California study [28]. For radon, we relied on a PAF developed for Ontario by Peterson et al [29] The radon PAF was based on the exposure-age-concentration BEIR-VI model to calculate the excess risk ratio relating indoor residential radon exposure to lung cancer mortality [30].

We developed de novo PAF estimates for solar ultraviolet (UV) radiation and second-hand smoke [14]. For second-hand smoke, Levin’s standard formula was adapted to incorporate the estimated number of lung cancer cases among non-smokers, which was calculated using a method described by Öberg et al [31] For solar UV radiation, we developed a PAF to estimate the number of UV radiation-attributable melanoma skin cancer cases using two methods to estimate melanoma incidence in an Ontario population unexposed to solar UV radiation. The first method, described by Parkin et al. [32], classified an Ontario 1913 birth cohort as the unexposed population, while the second method, described by Armstrong and Kricker [33], classified the African-American population in the United States as the unexposed population.

Incidence data for site-specific cancers in Ontario during the year 2011 were obtained from the Ontario Cancer Registry (Cancer Care Ontario SEER* Stat Package Release 10 – OCR [August 2015]). This data source recorded 9663 lung cancer cases and 3184 melanoma skin cancer cases in Ontario during the year 2011.

## Results

Of the 115 potential environmental carcinogen-exposure pathway pairings (23 × 5), we estimated annual cancer cases for 55 pairings of the 23 carcinogens and relevant routes of exposure (Table 4). Plausible pairings between environmental carcinogen and route of exposure were assessed (e.g., second-hand smoke in indoor air versus food) if sufficient input data were available (e.g., exposure and potency estimate). We reported estimates exceeding 10 annual mean cancers per year (our reporting threshold). Exposure to 3/20 carcinogens from outdoor air or sunlight exposures resulted in more than 10 annual mean cancer cases. Similarly, exposure to 5/18 indoor air carcinogens and 3/5 food carcinogens exceeded our reporting threshold. None of the annual mean cancers estimates exceeded the reporting threshold for drinking water (0/8) or dust (0/4). Data limitations were identified for food (3/8), drinking water (5/13), and indoor dust pairings (1/5).

**Table 4:**
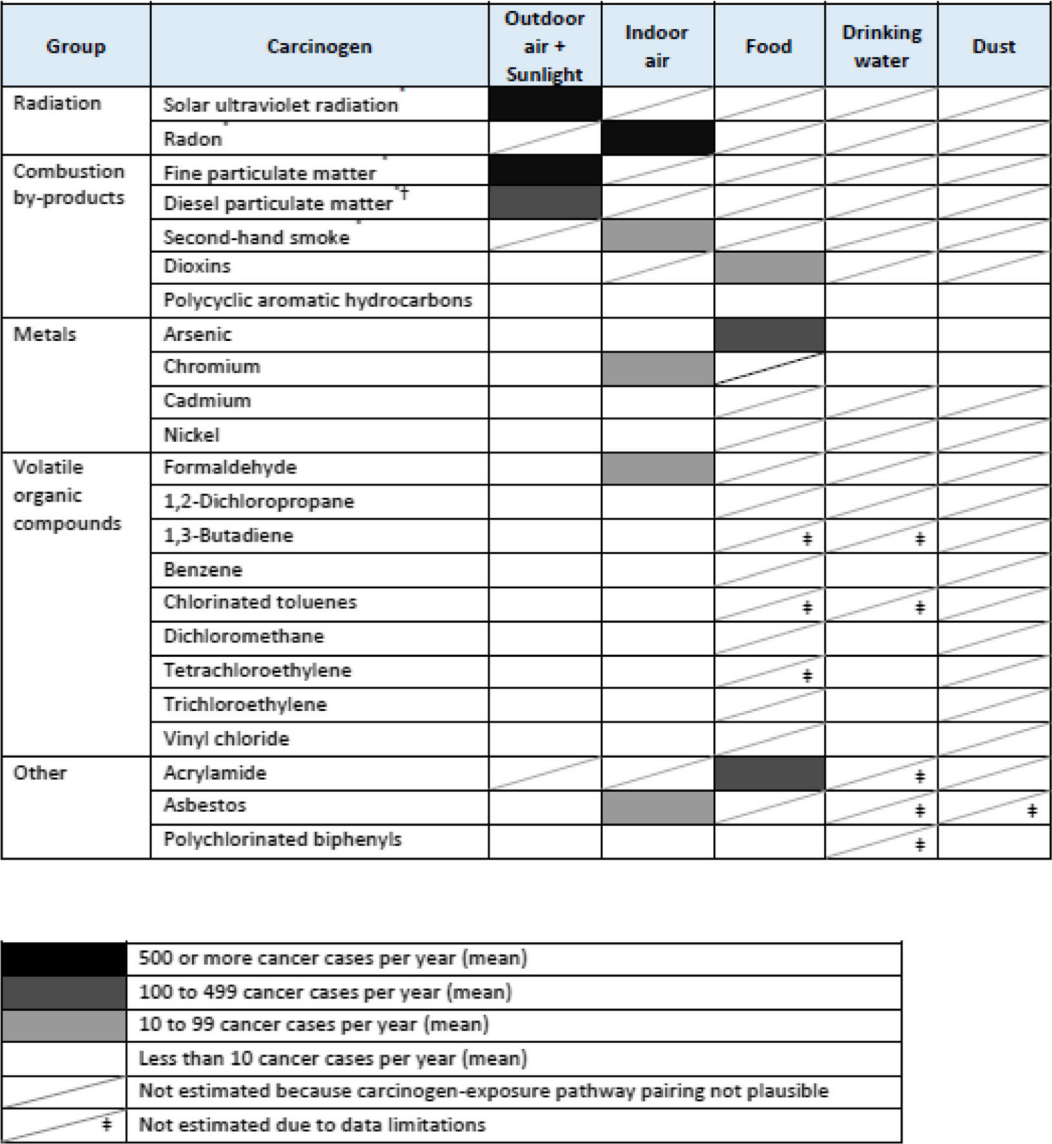
Mean estimated annual number of cancer cases by carcinogen and routes of exposure

^*^ Indicates a population attributable fraction model was used to estimate the annual cancer cases; otherwise a risk assessment model was used

^†^Diesel particulate matter was treated as a component of fine particulate matter (PM_2.5_), so the annual cancer cases should not be summed

We summed annual cancer case estimates across the routes of exposure for each carcinogen. Table 5 displays the simulation results for 10 of the 11 carcinogens meeting the reporting threshold (since diesel PM_2.5_ was treated as a subset of PM_2.5_, we do not present it in Table 5). The boxplot indicates the median, 25th and 75th percentile estimates by vertical lines, the mean with an x, and outliers (points outside 1.5 interquartile ranges of the 25th and 75th percentiles) with circles. The top three carcinogens -- solar UV radiation, radon, and PM_2.5_ – had mean annual cancer estimates over 500 cases and together accounted for over 90% of the environmental burden of cancer for the province. Arsenic and acrylamide had mean annual cancer estimates that were over 100 cases. Other carcinogens meeting the reporting threshold (in descending order) were: asbestos, formaldehyde, second-hand smoke, and dioxins. Overall, we estimated between 3540 and 6510 annual cancer cases in Ontario are attributable to environmental carcinogens.

**Table 5:** Estimated annual number of cancer cases from select environmental carcinogens in Ontario (rounded to nearest 10)

Carcinogen	Mean	Range	
		Lower estimate	Upper estimate
Solar ultraviolet (UV) radiation	2540	2090	2990
Radon	1310	1080	1550
Fine particulate matter (PM_2.5_)	560	290	900
Arsenic	120	20	370
Acrylamide	110	10	320
Asbestos	40	0	130
Formaldehyde	40	10	100
Second-hand smoke (SHS)	40	20	50
Dioxins	20	10	50
Chromium	10	0	20
TOTAL	4790	3540	6510

Only carcinogens with a mean annual burden above 10 are listed in the table

## Discussion

We estimated the annual number of cancer cases from exposure to 23 environmental carcinogens in Ontario. Understanding the relative contributions of these carcinogens to cancer burden may be useful in informing exposure reduction strategies and policy interventions. Regulations reducing emissions and setting tighter standards for food and consumer products have been used to reduce exposures to carcinogens of human origin. While this strategy is less useful in dealing with naturally occurring carcinogens, there is evidence that interventions targeting behavioural change can be effective in reducing these risks. For example, television advertising and internet education campaigns have increased sun-protection behaviors in Australia [34, 35] and the United States [36], respectively.

This analysis can be reproduced in the future to track trends, or be undertaken by other jurisdictions. The results provide estimates for the province as a whole and not for subregions or individual communities. However, much of the policy to address environmental carcinogens is made at the provincial level and we believe our estimates will provide a useful basis for priority setting by policy makers in both the health and environmental sectors.

Our estimate of approximately 3500 to 6500 annual cancer cases from exposure to environmental carcinogens in Ontario is larger than estimated cancer burdens from alcohol (1000 to 3000 cases) [37], obesity (2600 cases) [38], or infections (2400 to 3600 cases) [39]. However, our estimate is about half the cancer burden from tobacco smoking for the province (10,000 cases) [40]. Scaling annual estimates for the Canadian environmental burden of disease from Boyd and Genuis [41], who used an outcome-based approach to estimate burden, to the province of Ontario yields a range of 3200 to 9600. Their larger upper bound estimate may reflect their use of different input data sources, including previous burden of disease studies, to estimate an overall PAF.

Solar UV radiation, radon, and PM_2.5_ – in that order - dominated the estimated environmental burden of cancer in Ontario, with solar UV radiation accounting for the greatest number of cancer cases. In agreement with this finding, an assessment of cancer burden in the United Kingdom in 2010, for which the only overlapping carcinogens with our study were solar UV radiation, radon, and second-hand smoke, reported that malignant melanoma cases attributable to solar UV radiation were roughly an order of magnitude greater than lung cancer cases attributable to radon and lung cancer cases attributable to second-hand smoke [32, 42, 43]. Similarly, in an updated assessment of cancer burden in the United Kingdom in 2015, reported cancer cases attributable to solar UV radiation were roughly double those attributable to ionizing radiation (including radon) and roughly four-fold greater than cancer cases attributable to anthropogenic PM_2.5_ [8]. Hänninen and Knol [32] estimated the burden of cancer for five overlapping environmental carcinogens in six European countries. Their relative ranking (in disability-adjusted life years, DALY) was PM_2.5_ (nearly 350,000 DALYs), radon (over 190,000 DALYs), dioxins (over 110,000 DALYs), second-hand smoke (nearly 20,000 DALYs), and benzene (less than 1000 DALYs).

Our identification of solar UV radiation, radon, and PM_2.5_ as dominant contributors to the environmental burden of cancer in Ontario is in keeping with the findings of a formative cancer burden estimate for the United States [44]. Although their work was limited by the state of the evidence regarding environmental carcinogens that existed at that time, Doll and Peto nevertheless identified sunlight, background ionizing radiation, and pollution (air, water, and food) as important cancer risk factors [44]. However, their approach did not estimate the relative contributions of individual environmental carcinogens to cancer burden, as we have done in our study. Much of the information we used to perform our more detailed assessment was not available when Doll and Peto completed their work.

We applied the PAF method to five carcinogens. While cancer incidence is expected to increase over time, the relative ranking of the carcinogens is not expected to change if the increase is similar for the two cancer types we examined (melanoma and lung). Our PAF range of 64.0 to 95.4% for melanoma skin cancer cases attributable to solar UV radiation exposure encompasses equivalent PAFs reported for other high-income countries [6, 8, 32, 45, 46]. All of these PAFs were based on the use of early twentieth century birth cohorts, African Americans, or the general United Kingdom population to approximate unexposed populations. However, Grundy et al. based their substantially lower PAF estimate of 12.5% for solar UV radiation in Alberta, Canada, on a minimum risk exposure level of no sunburn in lifetime [5]. Their approach implies a threshold for the carcinogenic effects of UV radiation.

Our PAF of 13.6% (90% PI: 11.2 to 16.0%) for lung cancer cases attributable to radon exposure was similar to the PAF of 16.6% (95% CI: 9.4 to 29.8%) reported for an assessment of cancer burden in Alberta, Canada in 2012 [47]. To estimate a PAF for Alberta, Canada, Grundy et al. [47] followed the same approach as Peterson et al. [29], which was the source of the Ontario-specific PAF we used in our study. For both studies, the PAF was based on an exposure-age-concentration model from the United States National Research Council [30].

We estimated a PAF of 5.8% (90% PI: 3.0 to 9.4%) for lung cancer cases attributable to PM_2.5_ exposure in Ontario. This estimate was comparable to air pollution-lung cancer PAFs of 1.9 to 5.7% reported for Alberta, Canada in 2012 [48] and 7.8% reported for the United Kingdom in 2015 [8]. Although the PAF reported for the United Kingdom was based on the same relative risk from Hamra et al. [27] we used in our study, the Alberta PAF reflected levels above 7.5 or 3.2 μg/m^3^ and was based on older risk estimates [49, 50]. All three PAFs were developed using annual average PM_2.5_ concentrations specific to the province or country for which the burden was estimated.

We applied a risk assessment model to estimate the burden of 19 carcinogens across several routes of exposure including inhalation (indoor and outdoor air) and ingestion (food, drinking water, and dust). The top environmental carcinogens identified by this method were arsenic, acrylamide, asbestos, formaldehyde, dioxins, and chromium. Our findings are consistent with previous studies that examined fewer carcinogens and routes of exposure. Vromman et al. identified arsenic as a high priority carcinogen and/or genotoxic contaminant in food [51]. Woodruff et al. found 75% of the cancer risk of 148 hazardous air pollutants was due to polycyclic organic matter, 1,3-butadiene, formaldehyde, benzene, and chromium [11].

Consistent with any cancer burden analysis, there are several limitations associated with our study. First, our characterization of population exposure was limited by available data. For example, there were more carcinogen-exposure pairings that lacked exposure data for drinking water (*n* = 5) and food (*n* = 3) than there were for indoor or outdoor air (where we did not identify any exposure data limitations). Furthermore, some carcinogens had much more data to develop the input concentration distributions (e.g., there were 39 PM_2.5_ monitors across the province), but others had more limited data (e.g., we located only one study reporting an intake for food for polycyclic aromatic hydrocarbons). The data sources we used reflected general population exposure, rather than highly exposed individuals. We did not consider potential differences in susceptibility to environmental carcinogens for different population sub-groups. In part, this was due to a lack of available data but also in recognition that much of the policy in this area is made at a provincial or national level.

Second, our results reflect the many assumptions we made to conduct this analysis. The assumptions reflect the application of toxicological evidence, the characterization of mean exposures, and lifetime exposure periods for the risk assessment model. For the PAF model, they reflect the application of epidemiological evidence, the characterization of mean exposures, and latency information. We also decided to assume current exposures continue ‘as is’ into the future as we thought this would be more informative for policy makers than if we based estimates on historical exposures that cannot be addressed by new control policies.

Third, because of data availability, we were unable to account for in our analysis if one carcinogen impacted more than one cancer type. Similarly, we did not account for when many carcinogens impacted one cancer type. To illustrate this, we used the PAF model to estimate the lung cancers attributable to exposure to radon, PM_2.5_, and second-hand smoke. One approach to combine carcinogen exposures for the same disease is the product of complements (e.g., Prüss-Üstün et al. 2016) [43]. Applying this approach would scale each of our results by 0.95, reducing the radon estimate by 60 annual cases, the PM_2.5_ estimate by 30 annual cases, and keeping the SHS estimate the same (rounded to the nearest 10). It would not affect the rank ordering of our overall results.

Fourth, while we accounted for the population variability and known uncertainty in the inputs, we were unable to completely separate the two in our analysis. Table 6 highlights and contrasts the variability and/or uncertainty associated with each input. It should be noted, there is more uncertainty associated with the relative risks, PAFs, and OSFs/IURs than we were able to quantify. Similarly, we were unable to quantify uncertainty associated with the concentration estimates, though we know this to exist. Our modeling of population exposure can be interpreted as representing maximal uncertainty. Future analyses should fully separate uncertainty from variability.

**Table 6:** Variability and uncertainty in probabilistic inputs

Input	RA^a^	PAF^b^	Variable across the population?	Uncertain?
**Potency values (OSF and IUR)**	√		Potency may vary across the population, but there was no information to characterize it for this analysis.	Yes, different agencies may set different potency values; this is captured in this analysis (see Table 1). However, potency values are themselves uncertain and that was not captured in this analysis.
**Exposure factors (e.g., ingestion rate, bodyweight)**	√		Yes, exposure factors vary within and across age bins. This is captured in this analysis (see Additional file 3).	Yes, but there was no information to characterize it in this analysis.
**Population**	√		No, one point estimate for the province (from 2011 Census).	Yes, but uncharacterized. Expect the Census number to be robust.
**Concentration**	√	√	Yes, the exposure concentration varies across the population and this is captured in this analysis. However, there may be additional variability that is not captured by the input data sources.	Yes, but there was no information to characterize it for this analysis.^c^
**Relative risk**		√	Yes, the RR could differ by sub-populations (e.g., by sex or age group). This captured in the analysis whenever possible for UV and SHS.	Yes, the analysis captured the statistical uncertainty in the RRs (e.g., 95% confidence intervals) whenever possible. However, there is additional uncertainty associated with the RRs and that was not captured in this analysis for UV, SHS, DPM, and PM_2.5_.
**Population attributable fraction**		√	The PAF varies across the population, and this is captured for radon.	Yes, though we were unable to characterize it outside of the statistical uncertainty associated with radon and bounding the PAF obtained by different approaches in the UV analysis.
**Cancer Incidence**		√	No, one estimate for the province (from 2011 Ontario Cancer Registry).	Yes, but uncharacterized. Expect the Ontario Cancer Registry estimate to be robust.

*DPM* diesel particulate matter, *IUR* inhalation unit risk (IUR), *OSF* oral slope factor, *PAF* population attributable fraction, *PM*_*2.5*_ fine particulate matter, *RA* risk assessment, *SHS* second-hand smoke, *UV* ultraviolet

^a^Refer to Table 1 for the list of environmental carcinogens corresponding to the RA model (*n* = 19) and Additional file 3 for model details

^b^Refer to Table 2 for the list of environmental carcinogens corresponding to the PAF model (*n* = 5) and Additional file 4 for model details

^c^In interpreting the results of the simulation, one can imagine that there is maximal uncertainty at every concentration (e.g., 100% of population exposed at 25th percentile concentration for 25th percentile simulation result)

Our study also has several strengths. We systematically assessed the burden of all relevant environmental carcinogens and routes of exposure. We started with IARC-identified carcinogens and restricted our assessment based on their relevance to Ontario and the availability of exposure and potency data. As such, we were able to compare the burden for environmental carcinogens with very different sources and routes of exposure. Our analysis considered environmental agents deemed by IARC as carcinogenic (Group 1) or probably carcinogenic (Group 2A) to humans. Had we restricted our analysis only to Group 1 agents, acrylamide would be dropped from our results, since evidence for this carcinogen was deemed to be sufficient in animals but limited in humans. On the other hand, had we expanded our analysis to include agents deemed as possibly carcinogenic to humans (Group 2B; e.g., perfluorooctanoic acid or PFOA, electromagnetic fields or EMF) or whose carcinogenicity was deemed not classifiable (Group 3; e.g., a particular phthalate), we might have expanded our results, but faced additional data limitations regarding potency and concentration inputs.

The variability and uncertainty in the inputs is reflected in the simulation results. Additional file 5 shows how the mean results would change if the minimum or maximum value of each input were used (in tornado plots). The range in the overall cancer estimate was driven largely by the uncertainty in the estimates for our top two carcinogens, UV and radon. For these carcinogens, this was a function of the uncertainty in the population attributable fraction. In a sensitivity analysis, using the low or high PAF input resulted in an annual cancer estimate that was ±18% of the mean (UV) or ± 19% of the mean (radon). For the third ranking carcinogen, PM_2.5_, using the low or high input resulted in an annual cancer estimate that was ±41% of the mean for PAF and 35% below or 47% above the mean for concentration.

For arsenic, the most influential inputs were: the oral slope factor (low/high inputs resulted in annual cancers that were 65% below or 125% above the mean), the concentration of arsenic in food (low/high inputs resulted in annual cancers that were 33% below or 70% above the mean), the fraction of arsenic that was inorganic (low/high inputs resulted in annual cancers that were ± 45% of the mean), followed by other inputs (e.g., bodyweight/ingestion rate by age group, concentration of arsenic in outdoor air or dust; low/high inputs resulted in annual cancers that were ± 8% of the mean). By adopting a probabilistic approach instead of a deterministic approach, we were able to avoid implausibly precise (and potentially upward biased) burden estimates resulting from calculations using upper bound point estimates [52].

We provided a comparison of the burden of 23 environmental carcinogens across Ontario. This work could be conducted on a regular basis or extended to other jurisdictions, to identify top contributors to cancer burden and track progress in burden reduction. It could also be expanded as new carcinogens are identified and new potency information becomes available (e.g., [53]). One potential area for expansion is to re-examine the IARC agent classifications and select the environmental carcinogens, as carcinogens have been added since we conducted this work. Future work could also explore additional burden metrics. Other burden metrics include deaths, hospitalizations, cost, and disability-adjusted life years (which reflect mortality and morbidity). Assuming solar UV radiation is associated with malignant melanoma and that radon and PM_2.5_ are associated with lung cancer, differences in mortality between these two cancers might result in a difference in carcinogen rankings depending on whether one uses incident cancers or fatal cancers as the metric. However, to the extent that cancer mortality is also a function of the availability and effectiveness of treatment, this could dilute the focus on primary prevention.

## Conclusions

We estimated the annual cancer cases from exposure to 23 environmental carcinogens in Ontario, Canada using probabilistic risk assessment and PAF models. The burden (between 3540 and 6510 annual cancer cases) fell between previous estimates for the burden of alcohol and tobacco use. Top carcinogens were solar UV radiation, radon, and PM_2.5_, followed by arsenic and acrylamide. These findings can inform policy making and priority setting in health and environmental protection. This analysis could be reproduced at a later date to track trends in Ontario and could also be applied to other jurisdictions.

## Supplementary information

**Additional file 1.**

**Additional file 2.**

**Additional file 3.**

**Additional file 4.**

**Additional file 5.**

## Data Availability

All raw data used in the study were publicly available. References have been provided for all raw data. All modeling inputs have been provided in the tables in the manuscript and Additional Files. The datasets used and/or analysed during the current study are available from the corresponding author on reasonable request.

## Abbreviations

DPM: Diesel engine exhaust particulate matter
IARC: International Agency for Research on Cancer
PAF: Population attributable fraction
PM_2.5_: Fine particulate matter
UV: Ultraviolet
90% PI: 90% prediction interval

## References

[1] World Health Organization. Cancer fact sheet. World Health Organization. 2018. http://www.who.int/news-room/fact-sheets/detail/cancer. Accessed 30 Aug 2018.

[2] Cancer Care Ontario. Ontario cancer statistics 2018 Report. Cancer Care Ontario 2018. https://www.cancercareontario.ca/en/statistical-reports/ontario-cancer-statistics-2018-report. Accessed 02 Aug 2019.

[3] Xie L, Semenciw R, Mery L (2015). Cancer incidence in Canada: trends and projections (1983-2032). Health Promot Chronic Dis Prev Can.

[4] Schottenfeld D, Beebe-Dimmer JL, Buffler PA, Omenn GS (2013). Current perspective on the global and United States cancer burden attributable to lifestyle and environmental risk factors. Annu Rev Public Health.

[5] Grundy A, Poirier AE, Khandwala F, Grevers X, Friedenreich CM, Brenner DR (2017). Cancer incidence attributable to lifestyle and environmental factors in Alberta in 2012: summary of results. CMAJ Open..

[6] Islami F, Goding Sauer A, Miller KD, Siegel RL, Fedewa SA (2018). Proportion and number of cancer cases and deaths attributable to potentially modifiable risk factors in the United States. CA Cancer J Clin.

[7] Parkin DM, Boyd L, Walker LC (2011). 16. The fraction of cancer attributable to lifestyle and environmental factors in the UK in 2010: summary and conclusions. Br J Cancer.

[8] Brown KF, Rumgay H, Dunlop C, Ryan M, Quartly F, Cox A (2018). The fraction of cancer attributable to modifiable risk factors in England, Wales, Scotland, Northern Ireland, and the United Kingdom in 2015. Br J Cancer.

[9] Whiteman DC, Webb PM, Green AC, Neale RE, Fritschi L, Bain CJ (2015). Cancers in Australia in 2010 attributable to modifiable factors: introduction and overview. Aust N Z J Public Health.

[10] Hänninen O, Knol AB, Jantunen M, Lim TA, Conrad A, Rappolder M, Carrer P, Fanetti AC, Kim R, Buekers J, Torfs R (2014). Environmental burden of disease in Europe: assessing nine risk factors in six countries. Environ Health Perspect.

[11] Woodruff T, Caldwell J, Cogliano V, Axelrad D (2000). Estimating cancer risk from outdoor concentrations of hazardous air pollutants in 1990. Environ Res.

[12] Kauppinen T, Toikkanen J, Pedersen D, Young R, Ahrens W, Boffetta P (2000). Occupational exposure to carcinogens in the European Union. Occ Environ Med.

[13] Setton E, Hystad P, Poplawski K, Cheasley R, Cervantes-Larios A, Keller CP, Demers PA (2013). Risk-based indicators of Canadians’ exposures to environmental carcinogens. Environ Health.

[14] Public Health Ontario, Cancer Care Ontario. Environmental burden of cancer in Ontario: technical supplement. Public Health Ontario, Cancer Care Ontario. 2016. https://www.publichealthontario.ca/en/eRepository/Environmental_Burden_of_Cancer_Technical_2016.pdf. Accessed 02 Aug 2019.

[15] Gakidou E, Afshin A, Abajobir AA, Abate KH, Abbafati C, Abbas KM (2017). Global, regional, and national comparative risk assessment of 84 behavioural, environmental and occupational, and metabolic risks or clusters of risks, 1990–2016: a systematic analysis for the global burden of disease study 2016. Lancet.

[16] Prüss -Üstün A, Wolf J, Corvalán C, Bos R, Neira M. Preventing disease through healthy environments: a global assessment of the burden of disease from environmental risks. World Health Organization. 2016. https://www.who.int/quantifying_ehimpacts/publications/preventing-disease/en/. Accessed 02 Aug 2019.

[17] McKay PD, Beckman RJ, Conover WJ (1979). Comparison of three methods for selecting values of input variables in the analysis of output from a computer code. Technometrics..

[18] United States Environmental Protection Agency. Guidelines for carcinogen risk assessment report no. EPA/630/P-03/001F. United States Environmental Protection Agency. 2005. https://www.epa.gov/risk/guidelines-carcinogen-risk-assessment. Accessed 02 Aug 2019.

[19] Health Canada. Federal contaminated site risk assessment in Canada, part V: guidance on human health detailed quantitative risk assessment for chemicals (DQRA_Chem_). Health Canada 2010. ISBN: 978-1-100-17671-0. http://publications.gc.ca/pub?id=9.694270&sl=0. Accessed 02 Aug 2019.

[20] World Health Organization & International Programme on Chemical Safety. Principles for the assessment of risks to human health from exposure to chemicals. World Health Organization. 1999. http://www.inchem.org/documents/ehc/ehc/ehc210.htm. Accessed 02 Aug 2019.

[21] Richardson GM, Stantec Consulting Ltd. 2013 Canadian exposure factors handbook. Stantec. 2013. http://www.usask.ca/toxicology/docs/cef. Accessed 02 Aug 2019.

[22] Richardson GM (1997). Compendium of Canadian human exposure factors for risk assessment.

[23] Statistics Canada. Table 17-10-0005-01, population estimates on July 1st, by age and sex. Statistics Canada 2019. https://www150.statcan.gc.ca/t1/tbl1/en/tv.action?pid=1710000501. Accessed 22 Nov 2018.

[24] Health Canada. Federal contaminated site risk assessment in Canada, part II: Health Canada toxicological reference values (TRVs) and chemical-specific factors, version 2.0. Health Canada. 2010. http://publications.gc.ca/pub?id=9.694269&sl=0. Accessed 02 Aug 2019.

[25] United States Environmental Protection Agency. National Center for Environmental Assessment Integrated Risk Information System. United States Environmental Protection Agency. 2019. https://www.epa.gov/iris. Accessed 01 Jul 2015.

[26] California Office of Environmental Health Hazard Assessment. Chemicals. 2019. https://oehha.ca.gov/chemicals. Accessed 01 Jul 2015.

[27] Hamra GB, Guha N, Cohen A, Laden F, Raaschou-Nielsen O, Samet JM (2014). Outdoor particulate matter exposure and lung cancer: a systematic review and meta-analysis. Environ Health Perspect.

[28] California Environmental Protection Agency Air Resources Board. Report to the Air Resources Board on the proposed identification of diesel exhaust as a toxic air contaminant, part A: exposure assessment. California Environmental Protection Agency Air Resources Board. 1998. http://www.arb.ca.gov/toxics/dieseltac/part_a.pdf. Accessed 02 Aug 2019.

[29] Peterson E, Aker A, Kim J, Li Y, Brand K, Copes R (2013). Lung cancer risk from radon in Ontario, Canada: how many lung cancers can we prevent?. Cancer Causes Control.

[30] National Research Council (1999). Health effects of exposure to radon: BEIR VI. Committee on health risks of exposure to radon.

[31] Öberg M, Jaakola M, Pruss-Ulstun A, Scheizer C, Woodward A (2010). Second-hand smoke: assessing the environmental burden of disease at national and local levels.

[32] Hänninen O, Knol A. European perspectives on environmental burden of disease: estimates for nine stressors in six European countries. National Institute for Health and Welfare 2011. https://www.julkari.fi/bitstream/handle/10024/79910/b75f6999-e7c4-4550-a939-3bccb19e41c1.pdf. Accessed 02 Aug 2019.

[33] Armstrong B, Kricker A (1993). How much melanoma is caused by sun exposure?. Melanoma Res.

[34] Dobbinson SJ, Volkov A, Wakefield MA (2015). Continued impact of SunSmart advertising on youth and adults’ behaviors. Am J Prev Med.

[35] Dobbinson SJ, Wakefield MA, Jamsen KM, Herd NL, Spittal MJ, Lipscomb JE (2008). Weekend sun protection and sunburn in Australia: trends (1987–2002) and association with SunSmart television advertising. Am J Prev Med.

[36] Heckman CJ, Darlow SD, Ritterband LM, Handorf EA, Manne SL (2016). Efficacy of an intervention to alter skin cancer risk behaviors in young adults. Am J Prev Med.

[37] Cancer Care Ontario. Cancer risk factors in Ontario: alcohol. Cancer Care Ontario. 2014.: https://www.cancercareontario.ca/en/statistical-reports/cancer-risk-factors-ontario-alcohol. Accessed 02 Aug 2019.

[38] Cancer Care Ontario. Cancer risk factors in Ontario: healthy weights, healthy eating and active living. Cancer Care Ontario. 2015. https://www.cancercareontario.ca/en/statistical-reports/cancer-risk-factors-ontario-healthy-weights-healthy-eating-and-active-living. Accessed 02 Aug 2019.

[39] Cancer Care Ontario. Burden of cancer caused by infections in Ontario. Cancer Care Ontario. 2018. https://www.cancercareontario.ca/en/statistical-reports/cancer-risk-factors-ontario-burden-cancer-caused-infections-ontario. Accessed 02 Aug 2019.

[40] Cancer Care Ontario. Cancer risk factors in Ontario: tobacco. Cancer Care Ontario. 2014. https://www.cancercareontario.ca/en/statistical-reports/cancer-risk-factors-ontario-tobacco-0. Accessed 02 Aug 2019.

[41] Boyd DR, Genuis SJ (2008). The environmental burden of disease in Canada: respiratory disease, cardiovascular disease, cancer, and congenital affliction. Environ Res.

[42] Parkin DM (2011). 2. Tobacco-attributable cancer burden in the UK in 2010. Br J Cancer.

[43] Parkin DM, Darby SC (2011). 12. Cancers in 2010 attributable to ionising radiation exposure in the UK. Br J Cancer.

[44] Doll R, Peto R (1981). The causes of cancer: quantitative estimates of avoidable risks of cancer in the United States today. J Natl Cancer Inst.

[45] Olsen CM, Wilson LF, Green AC, Bain CJ, Fritschi L, Neale RE (2015). Cancers in Australia attributable to exposure to solar ultraviolet radiation and prevented by regular sunscreen use. Aust N Z J Public Health.

[46] Wilson LF, Antonsson A, Green AC, Jordan SJ, Kendall BJ, Nagle CM (2018). How many cancer cases and deaths are potentially preventable? Estimates for Australia in 2013. Int J Cancer.

[47] Grundy A, Brand K, Khandwala F, Poirier A, Tamminen S, Friedenreich CM (2017). Lung cancer incidence attributable to residential radon exposure in Alberta in 2012. CMAJ Open.

[48] Poirier AE, Grundy A, Khandwala F, Friedenreich CM, Brenner DR (2017). Cancer incidence attributable to air pollution in Alberta in 2012. CMAJ Open..

[49] Pope CA, Burnett RT, Thun MJ, Calle EE, Krewski D, Ito K (2002). Lung cancer, cardiopulmonary mortality, and long-term exposure to fine particulate air pollution. JAMA..

[50] Ostro B, World Health Organization (2004). Outdoor air pollution: assessing the environmental burden of disease at national and local levels.

[51] Vromman V, Maghuin-Rogister G, Vleminckx C, Saegerman C, Pussemier L, Huyghebaert A (2014). Risk ranking priority of carcinogenic and/or genotoxic environmental contaminants in food in Belgium. Food Addit Contam Part A Chem Anal Control Expo Risk Assess.

[52] Finley B, Paustenbach D (1994). The benefits of probabilistic exposure assessment: three case studies involving contaminated air, water, and soil 1. Risk Anal.

[53] Woodruff TJ (2015). Making it real—the environmental burden of disease. What does it take to make people pay attention to the environment and health?. J Clin Endocrinol Metab.

