# Supplementary material for: An approach to estimating the environmental burden of cancer from known and probable carcinogens: application to Ontario, Canada
Source: BMC Public Health. 2020 Jun 26;20:1017. doi: 10.1186/s12889-020-08771-w (PMC7320572; doi:10.1186/s12889-020-08771-w)
Supplement: Supplementary file 1 — Additional file 1. [file 12889_2020_8771_MOESM1_ESM.pdf]

Additional File 1. Carcinogen Selection and Cancer Types

Environmental carcinogen selection was based on a comprehensive framework previously developed for occupational carcinogens in Europe (Kauppinen et al. 2000), and later adapted for environmental carcinogens in Canada (Setton et al. 2013). Both frameworks are themselves based on International Agency for Research on Cancer (IARC) classifications. IARC classifies agents on the basis of carcinogenicity after examining evidence related to human and animal carcinogenicity. IARC deems:

- Group 1 agents as "carcinogenic to humans" based on finding sufficient evidence in humans to establish causal relationships between the exposure and development of cancer,
- Group 2A agents as "probably carcinogenic to humans" on the basis of limited evidence in humans, but sufficient evidence in animals, and
- Group 2B agents as "possibly carcinogenic to humans" on the basis of limited evidence in humans and insufficient evidence in animals.

Setton et al. (2013) focused on Group 1, Group 2A and Group 2B environmental carcinogens present in outdoor air, indoor air, indoor dust, drinking water, and foods and beverages (they did not include dermal absorption exposures because of a lack of data). We followed their approach, but excluded Group 2B carcinogens because we did not feel comfortable providing attributable cancer estimates for agents with limited or insufficient carcinogenicity evidence in animals or humans, given all the other assumptions that must be made in such an assessment.

Specifically, to select the environmental carcinogens:

- First, we consulted IARC on March 10, 2015 to obtain the listing of 188 agents they classified as "carcinogenic to humans" (i.e., Group 1) or "probably carcinogenic to humans" (i.e., Group 2A) (International Agency for Research on Cancer, 2015).
- Second, we focused on the 52 Group 1 and 2A carcinogens where we deemed the exposure category to be "environmental" (excluding 136 carcinogens from other exposure categories, related to items like occupation, behavior, or diet; see Table 1 and below for these excluded exposure categories and carcinogens).
- Third, we then grouped some related carcinogens together (e.g., different wavelengths of UV radiation; see Table 2 for the groupings), reducing the number of carcinogens to 38.
- Fourth, we determined that the average Ontarian would be unlikely to be exposed to 14 carcinogens during normal daily activities, leaving 24 carcinogens.
- Fifth, we deemed there to be insufficient data to classify exposure to the general public to one carcinogen (silica). Ultimately, we included the 23 carcinogens most relevant to the Ontario population in our assessment.

Table 2 provides a listing of the 52 environmental carcinogens and how the final list of 23 carcinogens included in the analysis was reached.

Table 1. Number and percentage of IARC Group 1 and 2A carcinogens by exposure category

	Group 1		*Group 2A*		*Total*	
Exposure Category	*n*	*%*	*n*	*%*	*n*	*%*
Behavioural	2	*1.7*	0	*0*	2	*1.1*
Dietary Agents	8	*6.8*	3	*4.3*	11	*5.9*
Environmental	32	*27.1*	20	*28.6*	52	*27.7*
Hormones	6	*5.1*	1	*1.4*	7	*3.7*
Microbiological Agents	12	*10.2*	3	*4.3*	15	*8.0*
Occupational	29	*24.6*	27	*38.6*	56	*29.8*
Pharmacologic Agents	22	*18.6*	16	*22.9*	38	*20.2*
Radionuclides	7	*5.9*	0	*0*	7	*3.7*
**Total**	**118**	**100%**	**70**	**100%**	**188**	**100%**

Specific carcinogens in Table 1 are listed below.

BEHAVIOURAL (n=2)

1. Tobacco smoking
2. Ultraviolet-emitting tanning devices

DIETARY AGENTS (n=11)

1. Acetaldehyde associated with consumption of alcoholic beverages
2. Alcoholic beverages
3. Areca nut
4. Betel quid with tobacco
5. Betel quid without tobacco
6. N'-Nitrosonornicotine (NNN) and 4-(N-Nitrosomethylamino)-1-(3-pyridyl)-1-butanone (NNK)
7. Salted fish, Chinese-style
8. Tobacco, smokeless
9. IQ (2-Amino-3-methylimidazo[4,5-f]quinoline)
10. Mate, hot
11. Nitrate or nitrite (ingested) under conditions that result in endogenous nitrosation

HORMONES (n=7)

1. Diethylstilbestrol
2. Estrogen-only menopausal therapy
3. Estrogen therapy, postmenopausal (see Estrogen-only menopausal therapy)
4. Estrogen-progestogen menopausal therapy (combined)
5. Estrogen-progestogen oral contraceptives (combined)
6. Ethanol in alcoholic beverages
7. Androgenic (anabolic) steroids

MICRBIOLOGICAL AGENTS (n=15)

1. Aflatoxins
2. Clonorchis sinensis (infection with)
3. Epstein-Barr virus
4. Helicobacter pylori (infection with)
5. Hepatitis B virus (chronic infection with)
6. Hepatitis C virus (chronic infection with)
7. Human immunodeficiency virus type 1 (infection with)
8. Human papillomavirus types 16, 18, 31, 33, 35, 39, 45, 51, 52, 56, 58, 59
9. Human T-cell lymphotropic virus type I
10. Kaposi sarcoma herpesvirus
11. Opisthorchis viverrini (infection with)
12. Schistosoma haematobium (infection with)
13. Human papillomavirus type 68
14. Malaria (caused by infection with Plasmodium falciparum in holoendemic areas)
15. Merkel cell polyomavirus (MCV)

RADIONUCLIDES (n=7)

1. Fission products, including strontium-90
2. Neutron radiation
3. Phosphorus-32, as phosphate
4. Plutonium
5. Radioiodines, including iodine-131
6. Radionuclides, alpha-particle-emitting, internally deposited
7. Radionuclides, beta-particle-emitting, internally deposited

PHARMACEUTICAL AGENTS (n=38)

1. Aristolochic acid
2. Aristolochic acid, plants containing
3. Azathioprine
4. Busulfan
5. Chlorambucil
6. Chlornaphazine
7. Cyclophosphamide
8. Cyclosporine (see ciclosporin)
9. Etoposide
10. Etoposide in combination with cisplatin and bleomycin
11. Melphalan
12. Methoxsalen (8-methoxypsoralen) plus ultraviolet A radiation
13. Methyl-CCNU
14. MOPP and other combined chemotherapy including alkylating agents
15. Phenacetin
16. Phenacetin, analgesic mixtures containing
17. Semustine (see Methyl-CCNU)
18. Sulfur mustard
19. Tamoxifen
20. Thiotepa
21. Thorium-232 and its decay products
22. Treosulfan
23. Adriamycin
24. Azacitidine
25. Bischloroethyl nitrosourea (BCNU)
26. Chloramphenicol
27. 1-(2-Chloroethyl)-3-cyclohexyl-1-nitrosourea (CCNU)
28. Chlorozotocin
29. Cisplatin
30. 5-Methoxypsoralen
31. N-Methyl-N´-nitro-N-nitrosoguanidine (MNNG)
32. Nitrogen mustard
33. Procarbazine hydrochloride
34. Teniposide
35. Chloral
36. Chloral hydrate
37. N-Methyl-N-nitrosourea
38. Pioglitazone

OCCUPATIONAL (n=56)

Group 1

1. Acheson process, occupational exposure associated with
2. Acid mists, strong inorganic
3. Aluminium production
4. 4-Aminobiphenyl
5. Auramine production
6. Benzidine
7. Benzidine, dyes metabolized to
8. Bis(chloromethyl)ether; chloromethyl methyl ether (technical-grade)
9. Coal gasification
10. Coal-tar distillation
11. Coal-tar pitch
12. Coke production
13. Erionite
14. Ethylene oxide
15. Fluoro-edenite fibrous amphibole
16. Haematite mining (underground)
17. Iron and steel founding (occupational exposure during)
18. Isopropyl alcohol manufacture using strong acids
19. Leather dust
20. Magenta production
21. 4,4'-Methylenebis(2-chloroaniline) (MOCA)
22. Mineral oils, untreated or mildly treated
23. 2-Naphthylamine
24. Painter (occupational exposure as a)
25. 2,3,4,7,8-Pentachlorodibenzofuran
26. Rubber manufacturing industry
27. Shale oils
28. Soot (as found in occupational exposure of chimney sweeps)
29. ortho-Toluidine

Group 2A

30. Art glass, glass containers and pressed ware (manufacture of)
31. Carbon electrode manufacture
32. 4-Chloro-ortho-toluidine
33. Cobalt metal with tungsten carbide
34. Diethyl sulfate
35. Dimethylcarbamoyl chloride
36. 1,2-Dimethylhydrazine
37. Dimethyl sulfate
38. N-Ethyl-N-nitrosourea (ENU)
39. Hairdresser or barber (occupational exposure as a)
40. Indium phosphide
41. Petroleum refining (occupational exposures in)
42. Shiftwork that involves circadian disruption
43. Silicon carbide whiskers
44. Vinyl bromide
45. Vinyl fluoride
46. Glycidol
47. Methyl methanesulfonate
48. 6-Nitrochrysene
49. N-Nitrosodiethylamine
50. N-Nitrosodimethylamine
51. 2-Nitrotoluene
52. Non-arsenical insecticides (occupational exposures in spraying and application of)
53. 1,3-Propane sultone
54. Tetrafluoroethylene (TFE)
55. 1,2,3-Trichloropropane
56. Tris(2,3-dibromopropyl) phosphate

Table 2. Listing of IARC Group 1 and 2A carcinogens for the environmental exposure category (n=52), as well as whether the carcinogen appears in the report's final list or the reason for exclusion

IARC Agent	IARC Group	Report final list (or reason for exclusion)
Outdoor air pollution, particulate matter in	1	$PM_{2.5}$
Outdoor air pollution	1	
Biomass fuel, indoor emissions from household combustion of	2A	
Radon-222 and its decay products	1	Radon
Radium-224 and its decay products	1	
Radium-226 and its decay products	1	
Radium-228 and its decay products	1	
Benzo[*a*]pyrene	1	PAH
Cyclopenta[*cd*]pyrene	2A	
Dibenz[*a,j*]acridine	2A	
Dibenz[*a,h*]anthracene	2A	
Dibenzo[*a,l*]pyrene	2A	
Polychlorinated biphenyls	1	PCB
3,4,5,3',4'-Pentachlorobiphenyl (PCB-126)	1	
Polychlorinated biphenyls, dioxin-like, with a Toxicity Equivalency Factor (TEF) according to WHO	1	
Engine exhaust, diesel	1	DEE
1-Nitropyrene	2A	
Solar radiation	1	UV
Ultraviolet radiation	1	
Ionizing radiation (all types)	1	*Excluded (see note A)*
X- and Gamma-Radiation	1	
2,3,7,8-Tetrachlorodibenzo-*para*-dioxin	1	Dioxin
Arsenic and inorganic arsenic compounds	1	Arsenic
Asbestos	1	Asbestos
Benzene	1	Benzene
1,3-Butadiene	1	1,3-Butadiene
Cadmium and cadmium compounds	1	Cadmium
Chromium (VI) compounds	1	Chromium
Formaldehyde	1	Formaldehyde
1,2-Dichloropropane	1	Dichloropropane
Nickel compounds	1	Nickel
Tobacco smoke, second-hand	1	SHS
Trichloroethylene	1	TCE
Vinyl chloride	1	VC
Acrylamide	2A	Acylamide
alpha-Chlorinated toluenes (benzal chloride, benzotrichloride, benzyl chloride) and benzoyl chloride (combined exposures)	2A	Chlorinated Toluenes
Tetrachloroethylene (Perchloroethylene)	2A	PCE

IARC Agent	IARC Group	Report final list (or reason for exclusion)
Dichloromethane (Methylene chloride)	2A	DCM
Coal, indoor emissions from household combustion of	1	*Excluded (see note B)*
Beryllium and beryllium compounds	1	*Excluded (see note B)*
Wood dust	1	*Excluded (see note B)*
Silica dust, crystalline, in the form of quartz or cristobalite	1	*Excluded (see note C)*
Ethyl carbamate (Urethane)	2A	*Excluded (see note B)*
Bitumens, occupational exposure to oxidized bitumens and their emissions during roofing	2A	*Excluded (see note B)*
Captafol	2A	*Excluded (see note B)*
Creosotes	2A	*Excluded (see note B)*
Epichlorohydrin	2A	*Excluded (see note B)*
Lead compounds, inorganic	2A	*Excluded (see note D)*
Polybrominated biphenyls	2A	*Excluded (see note B)*
Styrene-7,8-oxide	2A	*Excluded (see note E)*
Frying, emissions from high-temperature	2A	*Excluded (see note B)*
Ethylene dibromide	2A	*Excluded (see note B)*

Notes:

A. Excluded because difficult to assess exposure and solar and ultraviolet radiation has a bigger impact than ionizing radiation.
B. Not relevant for general population environmental exposure in Ontario
C. Insufficient data to assess general population exposure in Ontario
D. Excluded because exposure to inorganic lead in the general Ontario population is unlikely and there is no way to estimate this given available exposure data sources. (We do not include general lead exposure since that is classified by IARC as Group 2B.)
E. Excluded because general population is exposed to styrene (which is IARC Group 2B) not this short-lived metabolite.

Cancer types/sites associated with exposure to carcinogens

IARC provides information on the cancer type or cancer site associated with an agent having limited or sufficient evidence of causing cancer in humans (summarized in Table 3). Lung is a common cancer site, associated (limited or sufficient evidence) with exposure to over half of the carcinogens included in our work. Other common cancer sites/types were liver (associated with five carcinogens), bladder (associated with four carcinogens), and leukaemias (associated with three carcinogens).

Table 3. Summary of environmental carcinogens and associated cancer sites for which there is sufficient or limited evidence of cancer risk in humans, as classified by the International Agency for Research on Cancer (IARC)

Environmental carcinogen	Cancer site(s)	
	Sufficient evidence	Limited evidence
alpha-Chlorinated toluenes and benzoyl chloride		Lung
Arsenic	Lung, urinary bladder, skin (primarily squamous cell carcinoma)	Liver, prostate, kidney
Asbestos	Larynx, lung, mesothelioma, ovary	Pharynx, stomach, colon and rectum
Benzene	Acute myeloid leukaemia, acute non-lymphocytic leukaemia	Other leukaemias and lymphomas
1,3-Butadiene	Haematolymphatic organs	
Cadmium	Lung	Prostate
Chromium (VI)	Lung	Nasal cavity and paranasal sinus
Dichloromethane		Liver, non-Hodgkin lymphoma
1,2-Dichloropropane	Liver (cholangiocarcinoma)	
Diesel engine exhaust	Lung	Urinary bladder
Formaldehyde	Nasopharynx, leukemia	Nasal cavity and paranasal sinus
Nickel	Nasal cavity and paranasal sinus, lung	
Outdoor air pollution	Lung	Urinary bladder (soot)
Polychlorinated biphenyls	Melanoma	Breast, non-Hodgkin lymphoma
Radon and other alpha-particle emitters	Lung	Leukaemia
2,3,7,8-Tetrachlorodibenzo-para-dioxin	All sites (combined)	Lung, soft-tissue sarcoma, non-Hodgkin lymphoma
Tetrachloroethylene		Urinary bladder
Tobacco smoke, second-hand	Lung	Pharynx, larynx
Trichloroethylene	Kidney	Liver, non-Hodgkin lymphoma
Solar ultraviolet radiation	Skin (melanoma, squamous cell carcinoma, basal cell carcinoma)	Lip, eye
Vinyl chloride	Liver (angiosarcoma and hepatocellular carcinoma)	

Note: IARC did not provide any human cancer site/type information for acrylamide or PAHs (either individual PAHs, such as benzo[a]pyrene, or as a group) due to inadequate evidence from studies of cancer in humans. (These agents are classified as carcinogenic to humans because of strong mechanistic evidence in exposure humans.)

References

International Agency for Research on Cancer. IARC monographs on the evaluation of carcinogenic risks to humans. List of classifications, Volumes 1:109. 2015. Available here: https://monographs.iarc.fr/list-of-classifications (accessed March 2015)

Kauppinen T, Toikkanen J, Pedersen D, Young R, Ahrens W, Boffetta P, Hansen J, Kromhout H, Blasco JM, Mirabelli D, de la Orden-Rivera V. Occupational exposure to carcinogens in the European Union. Occupational and environmental medicine. 2000 Jan 1;57(1):10-8.

Setton E, Hystad P, Poplawski K, Cheasley R, Cervantes-Larios A, Keller CP, Demers PA. Risk-based indicators of Canadians' exposures to environmental carcinogens. Environmental Health. 2013 Dec;12(1):15.
