# Supplementary material for: An approach to estimating the environmental burden of cancer from known and probable carcinogens: application to Ontario, Canada
Source: BMC Public Health. 2020 Jun 26;20:1017. doi: 10.1186/s12889-020-08771-w (PMC7320572; doi:10.1186/s12889-020-08771-w)
Supplement: Supplementary file 2 — Additional file 2. [file 12889_2020_8771_MOESM2_ESM.pdf]

# Additional File 2. Augmented Concentration Details

We fit concentration distributions to the strongest secondary sources we could locate (from the year 2010 and in Ontario, if possible), including data from governmental websites and the peer-reviewed literature. The availability of environmental concentration data varied by carcinogen and environmental source. Data from monitoring campaigns or studies with larger sample sizes, robust sampling protocols, and Ontario-specific information were preferred.

## Limit of detection

For environmental data, a common approach to treat concentration estimates below the limit of detection (LOD), also known as non-detects, is substitution (e.g., by replacing the non-detects by concentrations of 0, the LOD/2, the LOD/√2, or the LOD). Substituting values below the LOD is consistent with exposure assessment practices elsewhere (e.g., Health Canada, US Centers for Disease Control and Prevention) and was employed in our analyses.

We use the following guidelines when we are calculating summary parameters *from raw data*:

1. We ascertained the LOD of the provided data (inquiring about the LOD if it was not stated in the documentation accompanying the data).
2. If the entity providing the data (i.e., the data steward) reported a result, we used it, even if the result was below the stated LOD.
3. If the entity providing the data reported a result as "<LOD" or "<DL" or "ND" or "<MRL", we substituted this value with the LOD/√2 (approximately 0.7071×LOD).

Where we obtained concentration information from journal articles, we ascertained how the authors treated samples below the LOD based on information provided in the manuscript and supplementary material (if applicable).

The LOD levels are presented in Table 1

**Table 1. Limit of detection (LOD) information by carcinogen and environmental source**

| Carcinogen | Environmental Source | LOD | Units | Sample Size | Percent reported (%)^ | Percent below LOD (%)^ |
|---|---|---|---|---|---|---|
| **Combustion by-products** | | | | | | |
| **Outdoor air pollution ($PM_{2.5}$)** | Outdoor Air | NR | μg/$m^3$ | 12122 | 100% | |
| **2,3,7,8-Tetrachlorodibenzo-para-dioxin (TCDD)** | Outdoor Air | NR | pg of TEQ/$m^3$ | 79 | 100% | |
| | Food and Beverages$^x$ | NR | pg of TEQ/kg-day | | | |
| **Polycyclic Aromatic Hydrocarbons (PAHs)** | Outdoor Air | NR | ng/$m^3$ | 198 | 100% | 0% |
| | Indoor Air | | | | | |
| | Indoor Dust | 0.051 | μg/g | | 100% | |
| | Drinking Water | 1 | ng/L | 68 | 100% | |
| **Metals and metalloids** | | | | | | |
| **Arsenic** | Outdoor Air | 0.02-0.06 | ng/$m^3$ | 384 | 100% | 2% |
| | Indoor Air | NR | ng/$m^3$ | 502 | 99% | 1% |
| | Indoor Dust | 0.1 | μg/g | 1025 | 100% | |
| | Drinking Water | 1 | μg/L | 277 | 100% | 92% |
| **Cadmium** | Outdoor Air | 0.02-0.06 | ng/$m^3$ | 384 | 100% | 6% |
| | Indoor Air | NR | ng/$m^3$ | 502 | 74% | 26% |
| | Indoor Dust | 0.1 | μg/g | 1025 | 100% | |
| | Drinking Water | 0.5 | μg/L | 277 | 100% | 100% |
| **Chromium (VI)** | Outdoor Air | 0.19-0.34 | ng/$m^3$ | 384 | 100% | 21% |
| | Indoor Air | NR | ng/$m^3$ | 502 | 57% | 43% |
| | Indoor Dust | 0.5 | μg/g | 1025 | 100% | |
| | Drinking Water | 5 | μg/L | 277 | 100% | 100% |
| **Nickel** | Outdoor Air | 0.09-0.24 | ng/$m^3$ | 384 | 100% | 21% |
| | Indoor Air | NR | ng/$m^3$ | 502 | 48% | 52% |
| | Indoor Dust | 0.5 | μg/g | 1025 | 100% | |
| | Drinking Water | 2 | μg/L | 277 | 100% | 95% |
| **Volatile organic compounds (VOCs)** | | | | | | |
| **1,2-Dichloropropane** | Outdoor Air | NR | ng/$m^3$ | 661 | 100% | 100% |
| | Indoor Air | 0.02 | ng/$m^3$ | 3857 | 4% | 96% |
| | Drinking Water | 0.05 | μg/L | 342 | 100% | 100% |
| **1,3-Butadiene** | Outdoor Air | NR | ng/$m^3$ | 1076 | 100% | 100% |
| | Indoor Air | 0.043-0.055 | ng/$m^3$ | 884 | 100% | 7% |
| **Alpha-** | Outdoor Air | NR | ng/$m^3$ | 283 | 100% | 0% |

| Carcinogen | Environmental Source | LOD | Units | Sample Size | Percent reported (%)^ | Percent below LOD (%)^ |
|---|---|---|---|---|---|---|
| **chlorinated toluenes** | Indoor Air | 0.018-0.050 | ng/m$^3$ | 845 | 100% | 97% |
| **Benzene** | Outdoor Air | NR | µg/m$^3$ | 1174 | 100% | 0% |
| | Indoor Air | 0.07 | µg/m$^3$ | 3857 | 100% | 0% |
| | Drinking Water | 0.05 | µg/L | 342 | 100% | 100% |
| **Dichloromethane** | Outdoor Air | NR | µg/m$^3$ | 1122 | 100% | 0% |
| | Indoor Air | 0.081-0.089 | µg/m$^3$ | 884 | 100% | 0% |
| | Drinking Water | 0.2 | µg/L | 342 | 100% | 100% |
| **Formaldehyde** | Outdoor Air | NR | µg/m$^3$ | 164 | 100% | 0% |
| | Indoor Air | NR | µg/m$^3$ | 215 | 100% | |
| **Tetrachloroethylene (PCE)** | Outdoor Air | NR | µg/m$^3$ | 1174 | 100% | 0% |
| | Indoor Air | 0.01 | µg/m$^3$ | 3857 | 99% | 1% |
| | Drinking Water | 0.05 | µg/L | 342 | 100% | 0% |
| **Trichloroethylene (TCE)** | Outdoor Air | NR | µg/m$^3$ | 1161 | 100% | 0% |
| | Indoor Air | 0.01 | µg/m$^3$ | 3857 | 75% | 25% |
| | Drinking Water | 0.05 | µg/L | 342 | 100% | 0% |
| **Vinyl chloride (chloroethene)** | Outdoor Air | NR | µg/m$^3$ | 844 | 100% | 0% |
| | Indoor Air | 0.110-0.115 | µg/m$^3$ | 884 | 100% | |
| | Drinking Water | 0.05 | µg/L | 342 | 100% | 100% |
| **Other** | | | | | | |
| **Acrylamide** | Food and Beverages | 10 | µg/kg of food | | | |
| **Asbestos** | Outdoor Air | 0 | f/mL | 1678 | 100% | |
| | Indoor Air | 0 | f/mL | 3979 | 100% | |
| **Polychlorinated biphenyls (PCBs)** | Outdoor Air | NR | pg TEQ/m$^3$ | 78 | 100% | |
| | Indoor Air | NR | pg ΣPCB/m$^3$ | 10 | 100% | |
| | Indoor Dust | NR | ng ΣPCB/g | 10 | 100% | |

LOD: limit of detection; NR: not reported.
Note: Except for acrylamide, no LOD information was provided for the food intakes.
^The percent reported refers to the fraction of samples where a value was provided by the data steward. Percent below limit of detection (LOD) refers to the percent of samples that were below the stated LOD. In our analysis, we used all values provided by the data steward (even if they were below the stated LOD). When the data steward listed a value as below the LOD, we performed substitution
[x] Except for acrylamide, no LOD information was provided for the food intakes.

# Food intake details

The food intakes (in units of µg/kg-d) were obtained from the Total Diet Study as mean values for approximately 11 age bins. When male and female mean intakes were provided separately for each age bin, we averaged them. In an effort to attempt to characterize variability associated with the food intake estimates, we used the spread of measures from the ten age bins, noting this will underestimate true variability. For PAH, we obtained an estimate of intake (in ng/d) converted it to intake units of ng/kg-d by dividing by bodyweight.

**Table 2. Food intake discrete probability distributions for dioxin, acrylamide, arsenic, PCBs, and PAHs**

| 2,3,7,8-tetrachlorodibenzo-para-dioxin (TCDD) | | Acrylamide | | Arsenic | | Polychlorinated biphenyls (PCBs) | | Polycyclic aromatic hydrocarbons (PAHs) | |
|---|---|---|---|---|---|---|---|---|---|
| Exposure (pg of TEQ/kg-day) | Probability | Exposure (µg/kg-day) | Probability | Exposure (µg/kg-day) | Probability | Exposure (ng/kg-day) | Probability | Exposure (ng/d) | Probability |
| 0.440 | 0.188 | 0.157 | 0.113 | 0.365 | 0.188 | 1.625 | 0.188 | 10.000 | 0.030 |
| 0.535 | 0.313 | 0.187 | 0.250 | 0.420 | 0.002 | 1.950 | 0.313 | 30.050 | 0.265 |
| 0.710 | 0.250 | 0.211 | 0.013 | 0.440 | 0.002 | 2.545 | 0.250 | 50.050 | 0.310 |
| 0.890 | 0.100 | 0.248 | 0.250 | 0.490 | 0.003 | 2.920 | 0.100 | 70.050 | 0.265 |
| 1.520 | 0.088 | 0.288 | 0.150 | 0.530 | 0.250 | 4.820 | 0.088 | 90.050 | 0.080 |
| 1.880 | 0.002 | 0.356 | 0.063 | 0.545 | 0.100 | 5.180 | 0.002 | 110.050 | 0.040 |
| 1.930 | 0.002 | 0.442 | 0.063 | 0.575 | 0.313 | 5.240 | 0.002 | 130.050 | 0.000 |
| 2.100 | 0.003 | 0.597 | 0.063 | 0.630 | 0.003 | 5.500 | 0.003 | 150.050 | 0.010 |
| 2.390 | 0.050 | 0.609 | 0.038 | 0.830 | 0.050 | 7.160 | 0.003 | | |
| 2.450 | 0.002 | | | 0.940 | 0.088 | 7.410 | 0.050 | | |
| 2.710 | 0.003 | | | 2.920 | 0.002 | 7.940 | 0.002 | | |

# Carcinogen-specific information

We compiled additional information related to the concentration estimation for several of the carcinogens, as listed below.

## Arsenic

For food ingestion, we model the fraction of food that is inorganic As versus organic by a uniform distribution, with range of 0.13 and 0.40 based on three studies (Schoof et al., 1999; Xue et al., 2010; Yost et al., 1998) summarized in Figure 1.

**Figure 1. Range of arsenic in food that is inorganic**

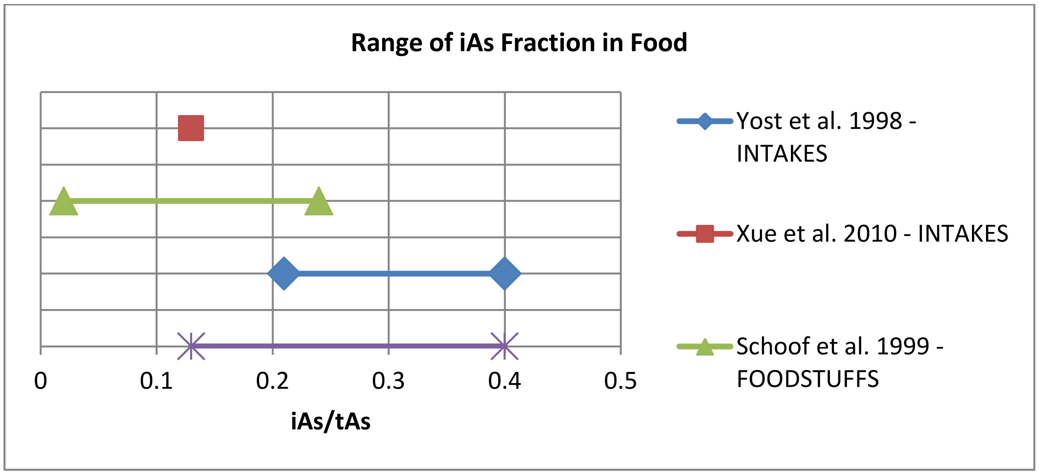


## Chromium

For Cr, we applied the fraction that is carcinogenic – that is the Cr(VI) fraction. We modelled this using a uniform distribution with a range of 0.1 to 0.5. We developed this range by evaluating information from a number of studies (Bell and Hipfner, 1997; Government of Canada, Health Canada, Environment Canada, 1994; Krystek and Ritsema, 2007; Mentze et al., 2004; Swietlik et al., 2011; Talebi, 2003; World Health Organization, 2003).

## Cadmium

While Cal EPA OEHHA lists a slope factor for cadmium ingestion, it is based on the inhalation unit risk. The WHO and USEPA do not classify cadmium as a carcinogen by the ingestion route of exposure. While IARC does not make a determination on the route of exposure, their association of cadmium with lung and prostate cancer is based on occupational studies where the primary route of exposure is inhalation. For this study, we estimated the cadmium cancer burden by inhalation and not ingestion.

## Diesel PM

No exposure estimates for diesel PM exist for Ontario. Such estimates are difficult to obtain for any region because of the measurement and modeling challenges. Instead, we derived a diesel PM distribution using data from a California Air Resources Board (CARB) report on identifying diesel exhaust as a toxic air contaminant (California Environmental Protection Agency Air Resources Board, 1998). For the year 1990, the CARB report estimated a statewide California population-weighted diesel $PM_{10}$ concentration of 3.0 µg/m$^3$, and projected this to decline to 1.7 µg/m$^3$ by the year 2010 (see Figure V-2 from CARB report). In other words, the 2010 diesel $PM_{10}$ level was 0.5667 times the 1990 level. In Table V-2 of the same CARB report, there are 1990 diesel $PM_{10}$ levels for 15 counties in California. We effectively converted these 1990 diesel $PM_{10}$ estimates to 2010 estimates by applying the 0.5667 factor. Next, we applied a diesel $PM_{2.5}/PM_{10}$ ratio of 0.94 (taken from another CARB report on the same issue, "The Report on Diesel Exhaust, Findings of the Scientific Review Panel On The Report on Diesel Exhaust") to effectively convert the diesel $PM_{10}$ estimates to diesel $PM_{2.5}$. Finally, we estimated an outdoor 2010 diesel $PM_{2.5}$ GM of 0.68 µg/m$^3$ (GSD of 2.35 µg/m$^3$) using the information above as applied to the 15 counties (Table 3).

Another approach to estimating the diesel PM levels would be to determine the fraction of $PM_{2.5}$ levels in Ontario that are of diesel exhaust origin. We did not locate any published values for this for Ontario. However, for six air basins in California, we have the 2010 diesel $PM_{2.5}$ estimates, along with monitored values for $PM_{2.5}$. For these six air basins, the fraction of $PM_{2.5}$ that was diesel ranged from 8% to 14%, with a mean value of 11% (median 11%; see Table 3). Applying 11% to the mean $PM_{2.5}$ level in Ontario (5.7 µg/m$^3$) gives an estimate of 0.57 µg/m$^3$, which is close to the modeled mean we employed of 0.67 µg/m$^3$. CAREX Canada has previously applied an estimate of 12% of $PM_{2.5}$ that is diesel $PM_{2.5}$, which is in line with our calculated estimate of 11%.

Furthermore, since the California Air Resources Board (CARB) report also stated that indoor levels of diesel are ⅔ of outdoor levels, we calculated an indoor level of 0.46 µg/m$^3$ (GSD of 2.35 µg/m$^3$) for the RA model.

Table 3. Lognormal 2010 diesel concentration distribution, fit to levels from 15 air basins

| Air Basin | Estimated Outdoor Diesel $PM_{2.5}$ (µg/m$^3$) | Measured Outdoor $PM_{2.5}$ (µg/m$^3$) | Diesel fraction (%) |
|---|---|---|---|
| *Great Basin Valley* | 0.11 | | |
| *Lake County* | 0.16 | | |
| *Lake Tahoe* | 0.53 | | |
| *Mojave Desert* | 0.43 | | |
| *Mountain Counties* | 0.32 | | |
| *North Central Coast* | 0.75 | | |
| *North Coast* | 0.64 | | |
| *Northeast Plateau* | 0.59 | | |
| *Sacramento Valley* | 1.33 | 10.9 | 12% |
| *Salton Sea* | 1.38 | | |

| | | | |
|---|---|---|---|
| *San Diego* | 1.54 | 10.8 | 14% |
| *San Francisco Bay Area* | 1.33 | 10.6 | 13% |
| *San Joaquin Valley* | 1.38 | 17.1 | 8% |
| *South Central Coast* | 0.96 | 10.0 | 10% |
| *South Coast* | 1.92 | 17.9 | 11% |
| *GM (µg/m$^3$)* | 0.68 | AM | 11% |
| *GSD (µg/m$^3$)* | 2.35 | Range | 8% - 14% |

AM: arithmetic mean; GM: geometric mean; GSD: geometric standard deviation

## Nickel

While we were able to calculate exposure concentrations from Ni in food, drinking water, and dust, there was no existing OSF. As such, we were unable to estimate the cancer burden by Ni ingestion. (We do estimate the cancer burden by Ni inhalation.)

## PAHs

PAHs represent a class of compounds. We used benzo[a]pyrene as a surrogate for total PAH exposure. While there are many other PAHs, benzo[a]pyrene is the most toxic. Health Canada, US EPA, and Cal EPA developed OSF and IUR estimates for benzo[a]pyrene, which we applied in our analysis.

## PCBs

PCBs are a class of compounds, consisting of many different congeners. There are two ways to treat this class of compound. One is to sum the individual PCB measurements and apply the PCB slope factor to this sum. Another is to weight the PCBs by their toxicities, summing the weighted values to obtain a toxic equivalency, or TEQ, then apply the dioxin slope factor to the TEQ. We applied the TEQ approach in our analysis.

References

Bell RW, Hipfner JC. Airborne hexavalent chromium in southwestern Ontario. J Air Waste Manage Assoc. 1997;47(8):905-10.

California Environmental Protection Agency Air Resources Board. Report to the Air Resources Board on the proposed identification of diesel exhaust as a toxic air contaminant. Part A: Exposure assessment [Internet]. Sacramento, CA: California Environmental Protection Agency Air Resources Board; 1998 [cited 2016 Jun 24]. Available from: http://www.arb.ca.gov/toxics/dieseltac/part_a.pdf

Government of Canada, Health Canada; Environment Canada. Canadian Environmental Protection Act: priority substances list assessment report. Chromium and its compounds. Ottawa, ON: Minister of Supply and Services Canada; 1994. Available from: http://www.hc-sc.gc.ca/ewh-semt/alt_formats/hecs-sesc/pdf/pubs/contaminants/psl1-lsp1/chromium_chrome/chromium_chrome-eng.pdf

Krystek P, Ritsema R. Monitoring of chromium species and 11 selected metals in emission and immission of airborne environment. Int J Mass Spectrom. 2007;265(1):23-9.

Metze D, Herzog H, Gosciniak B, Gladtke D, Jakubowski N. Determination of Cr (VI) in ambient airborne particulate matter by a species-preserving scrubber-sampling technique. Anal Bioanal Chem. 2004;378(1):123-8.

Schoof RA, Yost LJ, Eickhoff J, Crecelius EA, Cragin DW, Meacher DM, et al. A market basket survey of inorganic arsenic in food. Food Chem Toxicol. 1999;37(8):839-46.

Świetlik R, Molik A, Molenda M, Trojanowska M, Siwiec J. Chromium (III/VI) speciation in urban aerosol. Atmos Environ. 2011;45(6):1364-8.

Talebi SM. Determination of total and hexavalent chromium concentrations in the atmosphere of the city of Isfahan. Environ Res. 2003;92(1):54-6.

World Health Organization. Chromium in drinking-water: background document for development of WHO guidelines for drinking-water quality [Internet]. Geneva: World Health Organization; 2003 [cited 2016 Jun 24]. Available from: http://www.who.int/water_sanitation_health/dwq/chemicals/chromium.pdf28

Xue J, Zartarian V, Wang SW, Liu SV, Georgopoulos P. Probabilistic modeling of dietary arsenic exposure and dose and evaluation with 2003-2004 NHANES data. Environ Health Perspect. 2010;118(3):345. Available from: http://ehp.niehs.nih.gov/0901205/

Yost LJ, Schoof RA, Aucoin R. Intake of inorganic arsenic in the North American diet. Hum Ecol Risk Assess. 1998;4(1):137-52.
