# Supplementary material for: An approach to estimating the environmental burden of cancer from known and probable carcinogens: application to Ontario, Canada
Source: BMC Public Health. 2020 Jun 26;20:1017. doi: 10.1186/s12889-020-08771-w (PMC7320572; doi:10.1186/s12889-020-08771-w)
Supplement: Supplementary file 3 — Additional file 3. [file 12889_2020_8771_MOESM3_ESM.pdf]

Additional File 3. Risk Assessment Model Details

In estimating the environmental burden of cancer in Ontario, we followed general human health risk assessment frameworks. Risk assessment-specific inputs and the equations are provided in this section.

Equations to estimate excess cancers

Excess Cancers from Environmental Carcinogen Exposure from All Environmental Sources - RA (Series 1)

Equation 1-A. Excess Lifetime Cancer Cases from Environmental Carcinogen Exposure from All Environmental Sources

$$\text{Lifetime Cases} = \text{Lifetime Cases}_{\text{Air}} + \text{Lifetime Cases}_{\text{Drinking Water}} + \text{Lifetime Cases}_{\text{Dust}} + \text{Lifetime Cases}_{\text{Food}}$$

$$\text{Unit} = [\text{cases}]$$

Equation 1-B. Excess Lifetime (Individual) Risk from Environmental Carcinogen Exposure - General

$$\text{Lifetime Risk} = \text{Concentration} \cdot \text{Slope Factor}$$

$$\text{Unit} = [\text{risk}] = [\text{concentration}]\ \frac{[\text{risk}]}{[\text{concentration}]}$$

Equation 1-C. Excess Lifetime Cancers (in Ontario) from Environmental Carcinogen Exposure – General

$$\text{Lifetime Cases} = \text{Lifetime Risk} \cdot \text{Population}$$

$$\text{Unit} = [\text{cases}] = [\text{risk}]\ [\text{people}]$$

Equation 1-D. Excess Annual Cancer Cases (in Ontario) from Environmental Carcinogen Exposure from All Environmental Sources – General

$$\text{Annual Cases} = \frac{\text{Lifetime Cases}}{\text{Lifetime of 80 years}} = \frac{\text{Concentration} \cdot \text{Slope Factor} \cdot \text{Population}}{\text{Lifetime of 80 years}}$$

$$\text{Unit} = \frac{[\text{cases}]}{[\text{year}]}$$

Excess Cancer for the Inhalation Route of Exposure - RA (Series 2)

Equation 2-A. Lifetime Excess Cases from Inhalation

$$\text{Lifetime Cases}_{\text{Air}} = \text{Lifetime Cases}_{\text{Indoor Air}} + \text{Lifetime Cases}_{\text{Outdoor Air}}$$

$$\text{Unit} = [\text{cases}]$$

Equation 2-B. Lifetime Excess Cases for *Indoor* Air Inhalation

$$\text{Lifetime Cases}_{\text{Indoor Air}} = \{\text{fraction of time}_{\text{Indoors}} \cdot \text{Concentration}_{\text{Indoor Air}} \cdot \text{Inhalation Unit Risk}\} \cdot \text{Population}$$

$$\text{Unit} = [\text{cases}] = \left[\frac{\mu\text{g}}{\text{m}^3}\right] \left[\frac{\text{risk}}{\frac{\mu\text{g}}{\text{m}^3}}\right] [\text{people}]$$

Equation 2-C. Lifetime Excess Cases for *Outdoor* Air Inhalation

$$\text{Lifetime Cases}_{\text{Outdoor Air}} = \{ (1 - \text{fraction of time}_{\text{Indoors}}) \cdot \text{Concentration}_{\text{Outdoor Air}} \cdot \text{Inhalation Unit Risk}\} \cdot \text{Population}$$

$$\text{Unit} = [\text{cases}] = \left[\frac{\mu\text{g}}{\text{m}^3}\right] \left[\frac{\text{risk}}{\frac{\mu\text{g}}{\text{m}^3}}\right] [\text{people}]$$

Excess Cancer for the Ingestion Route of Exposure - RA (Series 3)

Equation 3-A. Lifetime Excess Cases from Ingestion

$$\text{Lifetime Cases}_{\text{Air}} = \text{Lifetime Cases}_{\text{DrinkingWater}} + \text{Lifetime Cases}_{\text{Dust}} + \text{Lifetime Cases}_{\text{Food}}$$

$$\text{Unit} = [\text{cases}]$$

Equation 3-B. Lifetime Excess Cases - Drinking Water Ingestion

$$\text{Lifetime Cases}_{\text{Drinking Water}} = \sum_{i=1}^{6} \text{F}_i \frac{\text{Ingestion Rate}_{\text{DrinkingWater}_i}}{\text{Bodyweight}_i} \cdot \{\text{Concentration}_{\text{Drinking Water}} \cdot \text{Oral Slope Factor} \cdot \text{Population}\}$$

$$\text{Unit} = [\text{lifetime cases}] = \left[\frac{\text{mg}}{\text{L}}\right]\left[\frac{\text{risk}}{\frac{\text{mg}}{\text{kg}-\text{d}}}\right]\left[\frac{\frac{\text{L}}{\text{d}}}{\text{kg}}\right][\text{people}]$$

Equation 3-C. Lifetime Excess Cases - Dust Exposure Ingestion

$$\text{Lifetime Cases}_{\text{Dust}} = \sum_{i=1}^{6} \text{F}_i \frac{\text{Ingestion Rate}_{\text{Dust}_i}}{\text{Bodyweight}_i} \cdot \{\text{Concentration}_{\text{Dust}} \cdot \text{Oral Slope Factor} \cdot \text{Population}\}$$

$$\text{Unit} = [\text{lifetime cases}] = \left[\frac{\text{mg}}{\text{mg}}\right]\left[\frac{\text{risk}}{\frac{\text{mg}}{\text{kg}-\text{d}}}\right][\frac{\frac{\text{mg}}{\text{d}}}{\text{kg}}][\text{people}]$$

Equation 3-D. Lifetime Excess Cases - Food Exposure Ingestion

$$\text{Lifetime Cases}_{\text{Food}} = \{\text{Intake}_{\text{Food}} \cdot \text{Oral Slope Factor}\} \cdot \text{Population}$$

$$\text{Unit} = [\text{lifetime cases}] = \left[\frac{\text{mg}}{\text{kg}-\text{d}}\right]\left[\frac{\text{risk}}{\frac{\text{mg}}{\text{kg}-\text{d}}}\right][\text{people}]$$

F_i represents the fraction of the lifespan spend in one of i=6 age bins with cut points at 1, 4, 12, 20, 65, and 80 years of age. Exposure factors for inhalation rate, drinking water ingestion rate, indoor dust ingestion rate, and bodyweight are available for the six age bins.

Note: We assume the bioavailability of carcinogens in food/dust/water is 100%.

Slope factor identification and inputs

We require a "dose-response" estimate, which provides a relationship between lifetime excess cancer risk and exposure for our analysis. For the RA, this takes the form of an OSF or IUR.

An **oral slope factor (OSF)** is an estimate of the increased cancer risk from oral exposure to a dose of, for example, 1 mg/kg-day for a lifetime. (*In our analysis, the OSF will be employed in the cancer EBD estimates for food, drinking water, and indoor dust ingestion.*) While a drinking water unit risk (DWUR) could be applied to the concentration in water directly to estimate lifetime risk to a carcinogen, we decided not to employ it in our analysis since DWURs are often calculated from OSFs, using default assumptions of 70 kg bodyweight and 2 L/day ingestion of water. Since we have exposure factors specific to the Canadian population in six age bins, we will apply these to the OSF for drinking water risks

An **inhalation unit risk (IUR)** is an estimate of the increased cancer risk from inhalation exposure to a concentration of, for example, 1 mg/m^3 for a lifetime. (*In our analysis, the IUR was employed in the cancer EBD estimates for the indoor and outdoor air inhalation.*)

The OSF and the IUR can be multiplied by an estimate of lifetime exposure (of dose in mg/kg-day or air concentration in mg/m^3, respectively) to estimate lifetime cancer risk. The slope factors are generally determined from fitting statistical models to animal or human occupational dose-response data, making assumptions, and using upper rather than mean model estimates of the relationship between dose and response.

We have collected OSFs and IURs for environmental pollutants derived and reported by the following agencies:

- Health Canada (HC)
- U.S. Environmental Protection Agency (USEPA)
- California EPA, Office of Environmental Health Hazard Assessment (OEHHA)

Generally, one particular study ("the critical effect study") forms the basis of the dose-response relationship where the oral slope factor or inhalation unit risk is derived. This study relates exposure to a particular carcinogen with the risk of developing a particular type of cancer.

We used a discrete uniform probability distribution to model the IURs and OSFs in @RISK. In other words, if one agency provided an estimate for the IUR (or OSF) for a carcinogen, we applied that estimate (weighting it by 100%). If two agencies provided an estimate, we weighted each estimate by 0.5. In the few cases that three agencies provided an estimate, we weighted each estimate by 0.33. See Table 1 and Table 2 for the IUR and OSF estimates, as well as the cancer sites associated with each carcinogen, as defined by IARC. (The RR estimates for the carcinogens that were evaluated using a PAF approach.)

Table 1. Oral slope factors (by agency) and summary of cancer site associated with carcinogen exposure in critical effect study

Carcinogen*	Oral Slope Factor (per mg/kg-day)					
	Health Canada	Cancer/ Species	US EPA	Cancer/ Species	CalEPA	Cancer/ Species
Combustion by-products						
**Diesel engine exhaust**						
**2,3,7,8-Tetrachlorodibenzo-para-dioxin (TCDD)**					1.3E+05	liver / mouse
**Polycyclic Aromatic Hydrocarbons (PAHs)**	2.3E+00	gastric / mice	7.3E+00	gastric / mice	2.9E+00	gastric / mice
Metals and metalloids						
**Arsenic**	1.8E+00	bladder, lung, liver / human	1.5E+00	skin / human	9.5E+00	skin / human
**Cadmium**[x]						
**Chromium (VI)**					5.0E-01	stomach / mice
**Nickel**						
Volatile organic compounds (VOCs)						
**1,2-Dichloropropane**					3.6E-02	liver / mice
**1,3-Butadiene**					6.0E-01	lung / mice
**alpha-Chlorinated toluenes**			1.7E-01	thyroid / rats	1.7E-01	thyroid / rats
**Benzene**[#]	8.3E-02	lymphoma / rats, mice	5.5E-02	leukemia / human, occupational	1.0E-01	leukemia / human, occupational
**Dichloromethane (methylene chloride)**	7.9E-05	lung / rats, mice	2.0E-03	liver / mice	1.4E-02	lung / mice
**Formaldehyde**						
**Tetrachloroethylene (PCE)**			2.1E-03	liver / mice	5.4E-01	liver / mice
**Trichloroethylene (TCE)**	8.1E-04	renal / rats	4.6E-02	renal, liver, non-hodgkin's lymphoma / humans	5.9E-03	liver, lymphoma / mice
**Vinyl chloride (chloroethene)^**	2.6E-01	liver / rats	1.5E+00	liver / rats	2.7E-01	lung / mice
Other						

Carcinogen*	Oral Slope Factor (per mg/kg-day)					
	Health Canada	Cancer/ Species	US EPA	Cancer/ Species	CalEPA	Cancer/ Species
**Acrylamide**			5.0E-01	thyroid, tunica vaginalis mesotheliomas / rats	4.5E+00	central nervous system, thyroid, breast, uterus, oral / rats
**Asbestos**						
**Polychlorinated biphenyls (PCBs)**			2.0E+00	liver, bile ducts / rats	2.0E+00	liver / mice

#Where one agency presented a range for the slope factor, the high range from that agency was used.

^The "from birth" value was selected from US EPA IRIS.

*The burden for these carcinogens was estimated using the RA model. The potency estimates for the carcinogens using the PAF model are presented separately.

ˣWhile CalEPA presented an OSF for cadmium we did not employ it.

Table 2. Inhalation unit risk (by agency) and summary of cancer site associated with carcinogen exposure in critical effect study

Carcinogen*	Inhalation Unit Risk (per µg/m^3)					
	Health Canada	Cancer/ Species	US EPA	Cancer/ Species	CalEPA	Cancer/ Species
Combustion by-products						
**Diesel engine exhaust**					3.0E-04	Lung / humans, occupational
**2,3,7,8-Tetrachlorodibenzo-para-dioxin (TCDD)**					3.8E+01	
**Polycyclic Aromatic Hydrocarbons (PAHs)**	3.1E-05	Respiratory tract / hamsters			1.1E-03	Respiratory tract / hamsters
Metals and metalloids						
**Arsenic**	6.4E-03	Lung / humans	4.3E-03	Lung / humans, occupational	3.3E-03	Lung / humans, occupational
**Cadmium**	9.8E-03	Lung / humans	1.8E-03	Lung, trachea, bronchus / humans, occupational	4.2E-03	Lung / humans, occupational
**Chromium (VI)**	7.6E-02	Lung / Human	1.2E-02	Lung / Human	1.5E-01	Lung / Human
**Nickel**					2.6E-04	Lung /Human, occupational
Volatile organic compounds (VOCs)						
**1,2-Dichloropropane**					1.0E-05	hepatocellular adenoma, carcinomas /mice
**1,3-Butadiene**			3.0E-05	leukemia / humans, occupational	1.7E-04	lung / mice
**alpha-Chlorinated toluenes**					4.9E-05	thyroid / rats
**Benzene#**	3.3E-06	leukemia / human, occupational	7.8E-06	leukemia / human, occupational	2.9E-05	leukemia / human, occupational
**Dichloromethane (methylene chloride)**	2.3E-08	lung, liver / rats, mice	1.0E-08	lung, liver / mice	1.0E-06	lung / mice
**Formaldehyde**			1.3E-05	squamous cell carcinoma/ rats	6.0E-06	nasal squamous carcinoma / rats

Carcinogen*	Inhalation Unit Risk (per µg/m^3)					
	Health Canada	Cancer/ Species	US EPA	Cancer/ Species	CalEPA	Cancer/ Species
**Tetrachloroethylene (PCE)**			2.6E-07	liver / mice	5.9E-06	liver / mice
**Trichloroethylene (TCE)**	6.1E-07	testes (leydig cells) / rats	4.1E-06	renal, liver, non-Hodgkin's lymphoma / humans	2.0E-06	lung, liver, lymphoma / mice
**Vinyl chloride (chloroethene)^**			8.8E-06	liver / rats	7.8E-05	lung / mice
**Other**						
**Acrylamide**			1.0E-04	thyroid, tunica vaginalis mesotheliomas / rats	1.3E-03	central nervous system, thyroid, breast, uterus, oral / rats
**Asbestos**[x]			2.3E-01	lung, mesothelioma / humans, occupational	1.9E+00	lung, mesothelioma / humans, occupational
**Polychlorinated biphenyls (PCBs)**			1.0E-04	liver, bile ducts / rats	5.7E-04	liver / rats

[#]Where one agency presented a range for the slope factor, the high range from that agency was used.

^The "from birth" value was selected from US EPA IRIS.

*The burden for these carcinogens was estimated using the RA model. The potency estimates for the carcinogens using the PAF model are presented separately.

[x]The units for the asbestos IUR are per fibres/mL

Exposure factors

We used exposure factors in the RA model when the OSF was applied to the drinking water and indoor dust concentration estimates. Additionally, for PAH food ingestion, we also make use of the body weight exposure factor. We obtained age-resolved estimates of central tendency and spread for the following exposure factors:

- Drinking water ingestion rate (Richardson, 1997)
- Indoor dust ingestion rate (Richardson and Stantec Consulting Ltd, 2013)
- Bodyweight (Richardson and Stantec Consulting Ltd, 2013)

We have adopted the six age group bins for a life expectancy of 80 years.

These dust ingestion rates are based on 50% hard and 50% soft surface results (last column) (Wilson et al., 2013).

The drinking water ingestion rate was lognormally distributed with GM and GSD shown in Table 3. The dust ingestion rate and bodyweight were normally distributed with AM and ASD shown in Table 3. The normal distributions were left-truncated at zero in @RISK to avoid generating implausible (negative) input parameters.

Table 3. Exposure factor distributions for ingestion and bodyweight, by age group

		Drinking Water ingestion rates (L/d)		Dust Ingestion Rate (mg/d)		Bodyweights (kg)	
**Age Group**	**Fraction of Lifespan**	*GM*	*GSD*	*AM*	*ASD*	*AM*	*ASD*
**Infant**	0.013	0.25	1.84	36	130	8.1	2
**Toddler**	0.038	0.5	1.84	41	71	15.3	2.3
**Child**	0.100	0.72	1.49	32	59	35.2	14.9
**Teen**	0.100	0.86	1.73	2.2	3.6	65.2	14.5
**Adult**	0.563	1.32	1.65	2.6	4.2	76.5	15.8
**Senior**	0.188	1.49	1.43	2.6	4.2	73.6	13.9

AM: arithmetic mean; ASD: arithmetic standard deviation; GM: geometric mean; GSD: geometric standard deviation

References:

Richardson GM. Compendium of Canadian human exposure factors for risk assessment. Ottawa, ON: O'Connor Associates Environmental Inc.; 1997.

Richardson GM, Stantec Consulting Ltd. 2013 Canadian exposure factors handbook: life expectancy, body dimensions, inhalation, time-activity, and soil ingestion [Internet]. Saskatoon, SK: Toxicology Centre, University of Saskatchewan; 2013 [cited 2016 Jun 24]. Available from: http://www.usask.ca/toxicology/docs/cef

Wilson R, Jones-Otazo H, Petrovic S, Mitchell I, Bonvalot Y, Williams D, et al. Revisiting Dust dust and Soil soil Ingestion Rates Based on Hand-to-Mouth Transfer. Hum Ecol Risk Assess. 2013;19:158-188.

Lifetime

For the environmental burden of cancer estimates generated by the RA method, we assume that individuals are exposed 100% of the time. We also assumed an 80 year lifespan. Our base year is 2010,

so we tried to obtain environmental concentration data for this year. For population data, we used the nearest Census year: 2011.

We calculated the lifetime risk of cancer (per carcinogen per environmental source) for one individual over an 80 year lifespan. Then, we multiplied this risk by the Ontario population that is under 80 years of age. We assume that all Ontario residents under 80, regardless of their age, are exposed for 80 years. We assume that the exposure concentrations calculated using 2010 data (or available data that was closest to 2010) are applicable to past and future exposures.

Fraction of time spent indoors

Additionally, we refer to the Canadian Exposure Factors Handbook (2013) to estimate the fraction of time spent indoors (see their Table 8.1). This was employed for indoor and outdoor air inhalation in the RA model. Our mean estimate of 95.76% was calculated from an estimate of adult total time indoors of 1379 minutes per day. (There are 24*60 = 1440 total minutes in a day.)

The fraction of time spent indoors follows a normal distribution, with AM of 0.96 (and ASD of 0.08), was constrained between the values of 0 (all time spent outdoors) and 1.0 (all time spent indoors) to avoid generating implausible inputs. This fraction was applied to indoor air inhalation in the RA and (1 – this fraction) was applied to outdoor air inhalation in the RA.

Reference:

Richardson GM, Stantec Consulting Ltd. 2013 Canadian exposure factors handbook: life expectancy, body dimensions, inhalation, time-activity, and soil ingestion [Internet]. Saskatoon, SK: Toxicology Centre, University of Saskatchewan; 2013 [cited 2016 Jun 24]. Available from: http://www.usask.ca/toxicology/docs/cef

Population

We calculated the 2011 Ontario population that is younger than 80 years to be: 12,745,163 (Population estimates, 2015).

Reference:

Population Estimates, 2000–12, Ontario Ministry of Health and Long-Term Care, IntelliHEALTH Ontario, Date Extracted: 2015 May 21.

Assumptions

We made several assumptions in estimating excess annual cancers from exposure to the carcinogens. These are listed below, along with the potential bias resulting from the assumption.

- Ontario residents are exposed 100% of the time to all carcinogens in the analysis for 80 years.
  - Upward bias expected, since exposure is likely less than 24 hours a day, 7 days a week for the lifetime.
- The lifespan of all Ontarians is 80 years.
  - No upward or downward bias expected from this lifespan assumption which was required to estimate annual risks from lifetime risks. In Ontario (for those born in 2007 to 2009), the life expectancy of males is 79 and females is 84, which are both in line with our estimate.
- The bioavailability of carcinogens in food/dust/water is 100%.
  - Upward bias expected, since the bioavailability of carcinogens may be less than 100%. Note, we attempted to quantify the toxic components of arsenic and chromium in our analysis.
- The IUR or OSF values that we selected and created distributions from are applicable to the Ontario population.
  - Upward bias expected, since IURs and OSFs generally represent upper bounds on excess lifetime risk of cancer from lifetime exposure.
- The IUR or OSF values that we employed were developed using data from a specific study on a particular species (e.g., human - occupational, animal) and cancer endpoint, but are applicable to the Ontario population to estimate general “excess cancers”.
  - Bias could be upward or downward, since the species may be sensitive or the carcinogens may result in more than one cancer and IURs and OSFs only capture one.
- When one agency reported a range for an OSF or IUR, the upper end of the range was selected for the analysis (e.g., EPA IRIS for benzene).
  - Upward bias expected.
- When an OSF or IUR estimate was provided for a specific lifespan, the lifetime value was applied if possible (e.g., VC the “from birth” value was selected).
  - No downward or upward bias expected, but this demonstrates that our approach does not account for critical periods of exposure for some of the carcinogens.
