# Supplementary material for: An approach to estimating the environmental burden of cancer from known and probable carcinogens: application to Ontario, Canada
Source: BMC Public Health. 2020 Jun 26;20:1017. doi: 10.1186/s12889-020-08771-w (PMC7320572; doi:10.1186/s12889-020-08771-w)
Supplement: Supplementary file 4 — Additional file 4. [file 12889_2020_8771_MOESM4_ESM.pdf]

Additional File 4. PAF Models for UV, radon, PM (and its subset, DEE), and SHS

Originally, we planned to apply a RA model to all 23 carcinogens. However, we did not locate potency information in the form suitable (e.g., oral slope factor, inhalation unit risk) to apply the RA model for five carcinogens: UV, radon, PM (and its subset, DEE), and SHS. For these carcinogens, we were able to locate potency information of another form (e.g., relative risk) to estimate the population attributable fraction (PAF). With an estimate of PAF, the cancers attributable to exposure to the carcinogen can be calculated as the product of the PAF and the observed cancer incidence. This approach is often employed for environmental burden of disease estimates for health endpoints other than cancer and is similar to the approach to generate the Global Burden of Disease (GBD) estimates (e.g., see Lim et al. (2013)).

For the RA model, the potency estimates are derived from fitting models to dose-response data (generated in animal or human studies). However, for the PAF model, the cancer type is specified in the relative risk relationship or in the derivation of the PAF by comparing "expected" and "observed" cancers.

This section outlines the development of the PAF for the five carcinogens where we employed the PAF model: UV, radon, PM (and its subset, DEE), and SHS.

<u>Reference</u>:

Lim SS, Vos T, Flaxman AD, Danaei G, Shibuya K, Adair-Rohani H, et al. A comparative risk assessment of burden of disease and injury attributable to 67 risk factors and risk factor clusters in 21 regions, 1990–2010: a systematic analysis for the Global Burden of Disease Study 2010. Lancet. 2012;380(9859):2224-60.

UV

There are several challenges in estimating how much melanoma skin cancer is attributable to solar ultraviolet (UV) radiation exposure, including the lack of population-based data on duration and patterns of exposure, and the absence of a truly non-exposed population. Previous epidemiological studies have used various approaches to define a non-exposed population in order to estimate the cancers attributable to UV. We reviewed the literature and, based on the nature of melanoma incidence data available in Ontario, selected two PAF approaches that were

suitable. We focused solely on melanoma, the most fatal form of skin cancer. The Ontario Cancer Registry (OCR) does not contain information about the more common basal cell carcinoma (BCC) and squamous cell carcinoma (SCC) skin cancers diagnosed in Ontario, and no other reliable source of information on non-melanoma skin cancers exists in the province.

UV PAF Method 1: Classifying 1913 birth cohort as unexposed

The first approach was based on a method (Parkin et al., 2011) that estimated UV-attributable cases as the difference between the observed number of cases and the number expected with a theoretical minimum-risk exposure distribution. For our calculation, the minimum-risk exposure distribution was based on historical data: the estimated incidence rates for Ontarians born in 1913. This allowed us to fit an age-cohort model to the data to recreate age-specific incidence rates for age groups without observations in the OCR. (High-quality melanoma incidence data is available in the OCR beginning in 1980.) This was done by sex (male, female) and age group (15–24, 25–34, 35–49, 50–64 and 65+ years).

We selected the cohort born in 1913 as the reference (non-exposed) population. Using the estimated incidence rates for this cohort, we calculated the expected number of cases in 2011 if sun exposure was the same as it had been in the 1913 birth cohort. The difference between this number and the number of observed melanoma cases in Ontario in 2011 is the estimated number of UV-attributable melanoma cases (i.e., attributable cases). The observed melanoma cases in 2011, the attributable cases, and the PAF estimates are shown in Table 1.

Reference:

Parkin DM, Mesher D, Sasieni P. 13. Cancers attributable to solar (ultraviolet) radiation exposure in the UK in 2010. Br J CancerBritish. 2011;105 Suppl 2:S66-9. Available from: http://www.nature.com/bjc/journal/v105/n2s/full/bjc2011486a.html33.

Table 1. Melanoma cases diagnosed in 2011 in Ontario and those estimated to be attributable to UV exposure with corresponding PAF, based on Method 1

	Males		Females		Both sexes	
Age (years)	**Observed cases**	**Attributable cases (PAF)**	**Observed cases**	**Attributable cases (PAF)**	**Observed cases**	**Attributable cases (PAF)**
15-24	13	7.2 (55.1)	32	22.0 (68.8)	45	29.2 (64.8)
25-34	47	27.5 (58.5)	82	51.8 (63.2)	129	79.3 (61.5)
35-49	227	152.1 (67.0)	282	188.6 (66.9)	509	340.7 (66.9)
50-64	544	376.5 (69.2)	423	284.3 (67.2)	967	660.8 (68.3)
65+	946	590.8 (62.5)	588	337.7 (57.4)	1.534	928.5 (60.5)
Total	1,777	1,154.0 (64.9)	1,407	884.4 (62.9)	3,184	2,038.4 (64.0)

PAF: population attributable fraction (calculated as the attributable cases divided by the observed cases)

UV PAF Method 2: Classifying an African-American population as unexposed

The second approach was based on a method (Armstrong and Kricker, 1993) that used melanoma incidence in the African-American population in the U.S. as a proxy for incidence in the non-exposed white population. Because the source of our observed melanoma estimates, the OCR, does not contain information on race or ethnicity, we used incidence data for the U.S. black population from the SEER (Surveillance, Epidemiology and End Results) 18 registries as a proxy for incidence in the non-exposed Ontario population. The SEER program of the National Cancer Institute in the U.S. provides cancer incidence data from population-based cancer registries covering approximately 30 per cent of the population. SEER 18 melanoma incidence rates for 2011 were extracted by sex (male, female) and age group (15–24, 25–34, 35–49, 50–64 and 65+) and applied to the Canadian 2011 postcensal estimates of the Ontario population to obtain the expected number of melanoma cases if incidence rates for the SEER 18 black population were observed in Ontario. The difference between this number and the number of observed melanoma cases in Ontario in 2011 was taken as the estimated number of UV-attributable melanoma cases (i.e., attributable cases). The observed melanoma cases in Ontario in 2011, the attributable cases, and the PAF estimates are shown in Table 2.

Reference:

Armstrong BK, Kricker A. How much melanoma is caused by sun exposure? Melanoma research. 1993;3(6):395-401.

Table 2. Melanoma cases diagnosed in 2011 in Ontario and those estimated to be attributable to UV exposure with the corresponding PAF, based on Method 2

	Males		Females		Both sexes	
Age (years)	Observed cases	Attributable cases (PAF)	Observed cases	Attributable cases (PAF)	Observed cases	Attributable cases (PAF)
15-24	13	13.0 (100.0)	32	31.0 (97.0)	45	44.0 (97.9)
25-34	47	45.8 (97.5)	82	80.9 (98.6)	129	126.7 (98.2)
35-49	227	222.8 (98.2)	282	274.4 (97.3)	509	497.3 (97.7)
50-64	544	528.6 (97.2)	423	398.4 (94.2)	967	927.0 (95.9)
65+	946	907.3 (95.9)	588	536.5 (91.2)	1.534	1,443.8 (94.1)
Total	1,777	1,717.6 (96.7)	1,407	1,320.4 (93.8)	3,184	3,037.9 (95.4)

PAF: population attributable fraction (calculated as the attributable cases divided by the observed cases)

Modeling the PAF for UV in the probabilistic assessment

Based on the two methods outlined above, we modeled the PAF for UV and skin cancer as a uniform distribution with a range of 0.640 to 0.954.

Assumptions

- The cancer burden of UV radiation can be quantified by examining melanoma skin cancer, for which IARC has deemed there to be sufficient evidence in humans; other cancers including those with sufficient evidence (non-melanoma skin cancers) but with not enough information and those with limited evidence in humans (e.g., lip and eye) were not examined
- The 1913 birth cohort in Ontario and the African-American population covered in the SEER 18 registries are reflective of the “non-exposed” Ontario population.
- All of the observed attributable melanoma cases are due to solar UV radiation exposure.
- Accounting for non-melanoma skin cancers (such as basal cell carcinoma and squamous cell carcinoma) would increase the number of skin cancers attributable to UV exposure. Non-melanoma skin cancers are also associated with UV exposure, however they are not included in these estimates of melanoma skin cancers since there is not a readily available non-melanoma skin cancer incidence estimate for Ontario (these typically treatable cancers are not tracked and it is difficult to develop an incidence estimate).

Radon

The impact of radon exposure in homes on the lung cancer burden in Ontario was recently estimated (Peterson et al., 2013). This study applied the method developed by Brand et al. who made use of an exposure-age-concentration model called BEIR-VI (Brand et al., 2005) to estimate the lung cancer burden of radon in Canada. Peterson et al. (2013) estimated the PAF (National Research Council, 1999) using Ontario data, separately for never- and ever-smokers to reflect the influence of smoking on lung cancer incidence.

The data sources and methods for Peterson et al. are reviewed in brief below and are described in more detail in the above references. To estimate radon exposure, the authors used Health Canada’s Cross-Canada Survey of Radon Concentrations in Homes. This survey was conducted from 2009 to 2011 and sampled 3,891 homes across Ontario. Radon exposure in Ontarians was found to follow a log-normal distribution with a GM of 43 Bq/m^3 and GSD of 3.1 Bq/m^3. (The radon exposure detection limit was 15 Bq/m^3.) The authors gathered data on factors that would influence radon exposure and lung cancer incidence, including the presence of apartment buildings (from Statistics Canada) and on smoking status (from Canadian Community Health Survey). The all-cause and lung cancer mortality information was derived from intelliHEALTH Ontario (year 2007).

As outlined in Brand et al. (2005), the BEIR-VI model was used to calculate the excess risk ratio (ERR) of lung cancer mortality (using a Monte Carlo simulation to assess uncertainty). Separately, life-table calculations were performed to determine the lifetime risk of lung cancer (LR) for ever- and never-smokers. The ERRs were used in the life-table calculations in order to determine the lifetime risk (LR_E) in radon-exposed individuals. Finally, the PAF was calculated using
$PAF = (LR_E - LR) / LR_E \times 100$.

Modeling the PAF for radon in the probabilistic assessment

From Table 1 of Peterson et al. (2013), the mean PAF estimate for radon and lung cancer in Ontario (combined for never- and ever-smokers) was 13.6% (median 13.5%), with a 95%CI of 11.0% to 16.7%. We modeled the PAF in @RISK using a normal distribution with mean of 13.6% and standard deviation of 1.45%. (We left-truncated this distribution at 0 and right-truncated it at 1.0 to avoid implausible results.)

References:

Brand KP, Zielinski JM, Krewski D. Residential radon in Canada: an uncertainty analysis of population and individual lung cancer risk. Risk Anal. 2005;25:253-269.

National Research Council. Health effects of exposure to radon: BEIR VI. Committee on health risks of exposure to radon. Washington, DC: National Academy Press; 1999. Available from: http://www.nap.edu/read/5499/

Peterson E, Aker A, Kim J, Li Y, Brand K, Copes R. Lung cancer risk from radon in Ontario, Canada: how many lung cancers can we prevent? Cancer Causes Control. 2013;24:2013-20. Available from: http://link.springer.com/article/10.1007%2Fs10552-013-0278-x

Assumptions

- The cancer burden of radon can be quantified by examining lung cancer, for which IARC has deemed there to be sufficient evidence in humans; other cancers with limited evidence in humans (e.g., leukaemia) were not examined.
- The exposure to radon for each public health unit in Ontario could be adequately modeling using data from Health Canada's Cross-Canada Survey of Radon Concentration in Homes, even though radon levels are known to vary widely from home to home and in some health units less than 100 samples were available.
- The estimated radon exposure is constant over a lifetime, though residential mobility is known to exist.
- The ever-smoker category (which included current, occasional, and previous smokers) to be the appropriate categorization for smoking risk, though this may be an oversimplification of the risk in this group. (It was employed to be consistent with the BEIR-VI model, a model used in the analysis.)

$PM_{2.5}$

We employed a PAF approach for $PM_{2.5}$ because there was no slope factor reported by the agencies we consulted. The PAF for $PM_{2.5}$ exposure (assuming 100% exposure prevalence) and lung cancer is:

$$\mathrm{PAF}_{\mathrm{Outdoor\ Air,PM}} = \left\{\frac{\mathrm{RR}-1}{\mathrm{RR}}\right\} = \left\{1 - \mathrm{e}^{-\beta\cdot\mathrm{Concentration}_{\mathrm{Outdoor\ Air,PM}}}\right\}$$

Where

RR is the relative risk where $\mathrm{RR} = \mathrm{e}^{\beta\cdot\mathrm{Concentration}_{\mathrm{Outdoor\ Air,PM}}}$

β is the slope derived from the study RR as $\frac{\ln(\mathrm{RR})}{\Delta\mathrm{X}}$

$\mathrm{Concentration}_{\mathrm{Outdoor\ Air,PM}}$ is the ambient $PM_{2.5}$ concentration

For the RR, we used the results from a recent analysis that was specifically designed to develop a quantitative estimate to accompany the IARC classification of $PM_{2.5}$ as a Group 1 carcinogen. Based on seven studies in North America (one of which was conducted in Canada), Hamra et al. (2014) conducted a random effects meta-analysis and reported a RR relating lung cancer incidence and $PM_{2.5}$ exposure of 1.11 (95% CI: 1.05, 1.16) per 10 μg/m^3 increase in $PM_{2.5}$. This RR corresponds to a β of 0.0104 (95% CI: 0.0049, 0.0148) per μg/m^3.

Modeling the PM PAF in the probabilistic analysis

For PM, the PAF was modeled using β in order to relate the potency with the PM levels. We modelled the β as a normal distribution in @RISK© with a mean of 0.0104 and a standard deviation of 0.0025 per μg/m^3, left-truncating the distribution at zero to avoid implausible estimates.

Assumptions

Assumptions for this approach include:

- The cancer burden of $PM_{2.5}$ can be quantified by examining lung cancer, for which IARC has deemed there to be sufficient evidence in humans; other cancers with limited evidence in humans (e.g., urinary bladder) were not examined
- Prevalence of exposure to $PM_{2.5}$ is 100%
- There is no threshold in the model (no PM level below which adverse effects would not occur); PM levels are compared to a level of 0 μg/m^3
- Annual average $PM_{2.5}$ concentrations from outdoor monitors reflect the appropriate concentration metric
- DEE is a subset of $PM_{2.5}$ and can be modeled using a RR developed for $PM_{2.5}$
- The RR from a meta-analysis of $PM_{2.5}$ environmental epidemiology studies is applicable to the Ontario population

Reference:

Hamra GB, Guha N, Cohen A, Laden F, Raaschou-Nielsen O, Samet JM, et al. Outdoor particulate matter exposure and lung cancer: a systematic review and meta-analysis. Environ Health Perspect. 2014;122(9):906-11. Available from: http://ehp.niehs.nih.gov/1408092/

Diesel $PM_{2.5}$

Diesel $PM_{2.5}$ is one component of ambient $PM_{2.5}$. As such, these estimates should be considered a portion of the ambient $PM_{2.5}$ estimates, and not added to them. Ambient PM is made up of primary PM (directly emitted) and secondary PM (formed from SO_2, NO_2, NH_3, and organics in the atmosphere) and has many sources (including natural – like volcanoes; and anthropogenic – like high temperature combustion from cars, trucks, buses, and power plants).

While we can also analyze diesel PM using a RA model, in our work we presented results using the PAF model to be consistent with the assessment model for $PM_{2.5}$. We did, however, compare the PAF model and RA model-derived estimates. We found that application of the PAF model for diesel PM resulted in a three-fold higher burden estimate than the RA model. This demonstrates that the two models will likely produce different estimates, but the same difference cannot be expected for other carcinogens even if the application of both models were possible.

SHS

We apply a PAF approach to estimate the proportion of incident lung cancer cases that can be attributed to exposure to environmental tobacco smoke (ETS)/second-hand smoke (SHS). Levin's standard formula was used:

$$\mathrm{PAF_{SHS}} = \frac{\mathrm{P_{SHS}}(\mathrm{RR_{SHS}} - 1)}{1 + \{\mathrm{P_{SHS}}(\mathrm{RR_{SHS}} - 1)\}}$$

Where
$\mathrm{PAF_{SHS}}$ is the proportion of incident lung cancer cases attributable to second-hand smoke exposure
$\mathrm{P_{SHS}}$ is the prevalence of second-hand smoke exposure at home among non-smokers and
$\mathrm{RR_{SHS}}$ is the relative risk of lung cancer for non-smokers exposed to second-hand smoke at home vs. non-smokers unexposed to second-hand smoke exposure at home.

Since the relative risk and prevalence estimates are based on non-smokers only, we needed to first estimate the number of new lung cancers among non-smokers as these statistics are not available from the Ontario Cancer Registry. To do this, we used the following method (Oberg et al., 2010):

Step 1. We calculated the proportion of new lung cancers attributable to current smoking (PAF_{CS}) using the following version of Levin's formula:

$$\mathrm{PAF_{CS}} = \frac{\mathrm{P_{CS}}(\mathrm{RR_{CS}} - 1)}{1 + \{\mathrm{P_{CS}}(\mathrm{RR_{CS}} - 1)\} + \{\mathrm{P_{FS}}(\mathrm{RR_{FS}} - 1)\}}$$

Where
$\mathrm{P_{CS}}$ = prevalence of current smokers,
$\mathrm{P_{FS}}$ = the prevalence of former smokers,
$\mathrm{RR_{CS}}$ = relative risk for current smokers vs. never smokers, and
$\mathrm{RR_{FS}}$ = relative risk for former smokers vs. never smokers.

Step 2. We calculated the number of lung cancer cases among non-smokers ($\mathrm{Lung\ Cancers_{NS}}$) by first subtracting the number of lung cancers attributable to current smoking from the total number of lung cancers diagnosed in Ontario during 2011 and then partitioning the resulting number of cancers according to the prevalence of non-smoking:

$$\mathrm{Lung\ Cancers_{NS}} = \{\mathrm{Total\ lung\ cancers} - (\mathrm{PAF_{CS}} \cdot \mathrm{Total\ lung\ cancers})\} \cdot \{1 - \mathrm{P_{CS}}\}$$

Once the number of lung cancers among non-smokers was estimated, we calculated the number of lung cancers due second-hand smoke exposures using the following equation:

$$\mathrm{Lung\ Cancers_{SHS}} = \mathrm{PAF_{SHS}} \cdot \mathrm{Lung\ Cancers_{NS}}$$

- The above steps were carried out and the PAF_{SHS} was calculated by sex (male, female) and age group (20-29, 30-44, 45-64, and 65+). The lung $cancers_{SHS}$ were summed for each age and sex group to get the total lung cancers attributable to SHS in Ontario.

- Prevalence data for second-hand smoke exposure at home among non-smokers and for current smoking was obtained from the 2009-2010 Canadian Community Health Survey (CCHS). Prevalence of exposure to second-hand smoke at home among non-smokers, as well as prevalence of current and former smoking, was calculated for Ontario by sex and age group. See Table 3.

Table 3. Prevalence (and standard deviation) of smoking for current and former smokers, as well as exposure to second-hand smoke in the home, by sex and age

	P_{CS}		P_{FS}		P_{SHS}	
	Prevalence	s.d.	Prevalence	s.d.	Prevalence	s.d.
Males						
20–29	0.3097	0.0169	0.0877	0.0079	0.1055	0.0126
30–44	0.2691	0.0117	0.2012	0.0093	0.0279	0.0074
45–64	0.2569	0.0101	0.3398	0.0109	0.0459	0.0060
65+	0.0938	0.0073	0.5357	0.0116	0.0328	0.0036
Females						
20–29	0.2203	0.0130	0.0981	0.0075	0.0566	0.0074
30–44	0.1640	0.0079	0.1717	0.0085	0.0352	0.0073
45–64	0.1761	0.0085	0.2758	0.0092	0.0379	0.0045
65+	0.0901	0.0051	0.3000	0.0085	0.0245	0.0034
Both sexes						
20–29	0.2651	0.0110	0.0929	0.0054	0.0796	0.0072
30–44	0.2157	0.0073	0.1862	0.0063	0.0319	0.0052
45–64	0.2161	0.0065	0.3074	0.0071	0.0416	0.0039
65+	0.0917	0.0042	0.4058	0.0070	0.0282	0.0025

P_{CS}: prevalence of current smokers; P_{FS}: prevalence of former smokers; P_{SHS}: prevalence of exposure to second-hand smoke in the home; s.d.: standard deviation
Data source: Canadian Community Health Survey 2009-10 (Statistics Canada, 2010)

- Relative risk estimates for the association between lung cancer and second-hand smoke exposure among non-smokers and for the association between lung cancer and current and former smoking were obtained from the literature. These estimates and their associated sources are outlined in Table 4. The relative risks for a second-hand smoke exposure among non-smokers and for former smoking vs. never smoking were assumed to be the same for males and females and for all age-groups. Sex-specific relative risks were used for current smoking vs. never smoking but within each sex the relative risks were assumed to be the same for all age-groups.

Table 4. Summary of relative risks employed in the second-hand smoke PAF Approach

Exposed Population vs. Referent Population	Relative Risk (95% CI)	
	Males	Females
Second-hand smoke at home among non-smokers vs. non -smokers unexposed at home(RR_{SHS})(Oberg et al., 2010)	1.21† (1.13, 1.30)	1.21† (1.13, 1.30)
Current smoking vs. never smoking (RR_{CS}) (Gandini et al., 2008)	9.87 (6.85, 14.24)	7.58 (5.36, 10.73)
Former smoking vs. never smoking (RR_{FS}) (Gandini et al., 2008)	3.85† (2.77, 5.35)	3.85† (2.77, 5.35)

CI: confidence interval; RR_{CS}: relative risk for current smokers; RR_{FS}: relative risk for former smokers; RR_{SHS}: relative risk for those exposed to second-hand smoke

† No difference in estimate of relative risk by sex

Modeling the SHS PAF in the probabilistic analysis

The prevalence estimates were modeled as normal distributions with the corresponding means and standard deviations in Table 3. The RRs were modeled as normal distributions, with the means as shown in Table 4 and the standard deviations calculated from the 95% CI. The mean estimates of the PAF for SHS ranged from 0.5% to 2.2% across the age and sex subgroups and was 0.6% overall (Table 5).

Table 5. Inputs and outputs of second-hand smoke PAF approach

Age (years)	Total lung cancers	Lung $cancers_{NS}$	Lung $cancers_{SHS}$ (PAF_{SHS})† Mean estimates
Males			
20–29	6	1	0 (2.2%)
30–44	48	14	0 (0.6%)
45–64	1,307	450	4 (1.0%)
65+	3,623	2,470	17 (0.7%)
Total, males	4,984	2,936	21 (0.7%)
Females			
20–29	6	2	0 (1.2%)
30–44	71	34	0 (0.7%)
45–64	1,423	711	6 (0.8%)
65+	3,175	2,190	11 (0.5%)
Total, females	4,674	2,937	17 (0.6%)
Both sexes			
20–29	12	3	0 (1.6%)
30–44	119	48	0 (0.7%)
45–64	2,730	1,161	10 (0.9%)
65+	6,798	4,660	28 (0.6%)
Total	9,658	5,872	38 (0.6%)

†Lung $cancers_{SHS}$ are calculated as the product of the Lung $cancers_{NS}$ and the PAF_{SHS} and rounded to the nearest whole number for the age and sex subgroups. For the total, the Lung $cancers_{SHS}$ are the sum of the age subgroup Lung $cancers_{SHS}$ and the PAF_{SHS} is estimated from the Lung $cancers_{SHS}$ divided by the Lung $cancers_{NS}$.
Data sources: Total lung cancers for year 2011 from Ontario Cancer Registry, 2015 (Cancer Care Ontario, 2019); Lung $Cancers_{NS}$, Lung $Cancers_{SHS}$, and PAF_{SHS} estimated from equations above, using prevalence data in Table 3 and relative risks from Table 4.

Assumptions

Assumptions for this approach include:

- The cancer burden of SHS can be quantified by examining lung cancer, for which IARC has deemed there to be sufficient evidence in humans; other cancers with limited evidence in humans (e.g., pharynx, larynx) were not examined
- Exposure to smoking in the home (rather than in "any location") is the relevant metric to capture the prevalence of exposure to SHS indoors
- Second-hand smoke exposure among the non-smoking population has not changed over time. Therefore, prevalence estimates from 2009-2010 assumed to be representative of past exposure.
- There is no lag time between exposure to second-hand smoke and the development of lung cancer.
- The study populations from which the relative risk estimates are derived are representative of the Ontario population and reflect the risk of lung cancer associated with second-hand smoke exposure at the present time.
- Second-hand smoke exposure does not influence the risk of developing lung cancer among current smokers and therefore no lung cancers among current smokers are attributable to second-hand smoke exposure.

Our assumptions related to current second-hand smoke prevalence being representative of past exposures and no lag time between exposure and disease are consistent with the assumptions we made for the other carcinogens and allow comparison across carcinogens. However, we acknowledge that exposure to second-hand smoke at home has declined significantly over the past decade and a lag time of 10 to 20 years between exposure and the development of lung cancer is more realistic. To examine the potential influence of these simplifying assumptions, we calculated the burden using prevalence estimates from 2000/01 (instead of 2009/10), thereby introducing an 11 year lag and found a central estimate of 68 cancers attributed to SHS compared to 38, or 1.8 times higher estimates.

References:

Cancer Care Ontario. Ontario Cancer Registry. Ontario: Cancer Care Ontario. 2019. Available from: https://www.cancercareontario.ca/en/cancer-care-ontario/programs/data-research/ontario-cancer-registry

Gandini S, Botteri E, Iodice S, Boniol M, Lowenfels AB, Maisonneuve P, et al. Tobacco smoking and cancer: a meta-analysis. Int J Cancer 2008;122(1):155-64. Available from: http://onlinelibrary.wiley.com/doi/10.1002/ijc.23033/full

Öberg M, Jaakkola MS, Prüss-Ülstün A, Scheizer C, Woodward A. Second-hand smoke: Assessing the environmental burden of disease at national and local levels. Geneva: World Health Organization; 2010. Available from: http://www.who.int/quantifying_ehimpacts/publications/SHS.pdf?ua=1

Statistics Canada. Canadian Community Health Survey - Annual Component (CCHS) Detailed information for 2009. Ottawa: Statistics Canada. 2010. Available from: http://www23.statcan.gc.ca/imdb/p2SV.pl?Function=getSurvey&Id=67251

Estimated population attributable fractions, relative risks, and slopes (PAF model)

The potencies for the PAF model are either from PAFs directly (radon and UV), from RRs (SHS), or from calculated measures from RRs ($PM_{2.5}$). See Table 6, which also notes the cancer site associated with the study for each carcinogen.

Table 6. Probabilistic inputs for the potencies for the PAF model

Carcinogen	Cancer site	Note	Metric	AM	ASD
**$PM_{2.5}$**#	Lung	Units: per μg/m^3	slope	0.0104	0.0025
**UV**^	Skin	Method 1	PAF	0.640	NA
		Method 2	PAF	0.954	NA
**Radon**	Lung		PAF	0.136	0.015
**Second-hand smoke**†	Lung	SHS	PAF	0.006	NA
		SHS	RR	1.21	0.04
		CS/male	RR	9.87	1.89
		CS/female	RR	7.58	1.37
		FS	RR	3.85	0.66

AM: arithmetic mean; ASD: arithmetic standard deviation; CS: current smoker; FS: former smoker; RR: relative risk; NA: not applicable; PAF: population attributable fraction; SHS: second-hand smoke
$PM_{2.5}$ slope also applied to diesel $PM_{2.5}$; units are per μg/m^3
^ The UV PAF was modeled as a uniform distribution, with the range as the AMs from Method 1 and 2

Incident Cancer Cases

We applied several point estimates in our probabilistic analysis, as defined in Table 7.

Table 7. Point estimates in probabilistic analysis

Parameter	Model	Value	Unit
Incident melanoma cases (2011)^	PAF	3,184	cases
Incident lung cancer cases (2011)^	PAF	9,663	cases

PAF: population attributable fraction; RA: risk assessment
†Data Source: Pop Est Summary (Statistics Canada, Ontario Ministry Finance), Fall 2014 release, based on the 2011 Census
^Data Source: CCO SEER*Stat Package Release 10 - OCR (Aug. 2015).

Probabilistic Analysis

A probabilistic analysis incorporates the variability and uncertainty in the inputs.

- **Variability**: Refers to true differences in attributes due to heterogeneity. Not usually reduced by further measurement/study, though it can be better characterized.
- **Uncertainty**: Lack of information. Uncertainty analysis attempts to describe the degree to which a calculated value may differ from a true value.

In our analysis, we characterized variability and uncertainty to the extent possible.

Error! Reference source not found. indicates the variability and uncertainty for each parameter, and if or how it was characterized in the analysis.
