# Supplementary material for: An approach to estimating the environmental burden of cancer from known and probable carcinogens: application to Ontario, Canada
Source: BMC Public Health. 2020 Jun 26;20:1017. doi: 10.1186/s12889-020-08771-w (PMC7320572; doi:10.1186/s12889-020-08771-w)
Supplement: Supplementary file 5 — Additional file 5. [file 12889_2020_8771_MOESM5_ESM.pdf]

Additional File 5. Tornado Plots Associated with Results

Tornado plots illustrate how the mean results might change when the low/high value of a particular input is used instead of the distribution for that input. Please note that these results correspond to the carcinogens shown in Figure 2 of the manuscript. Please also note that due to the nature of simulation modeling, the mean results below will not match exactly the mean results in Figure 2 of the manuscript.

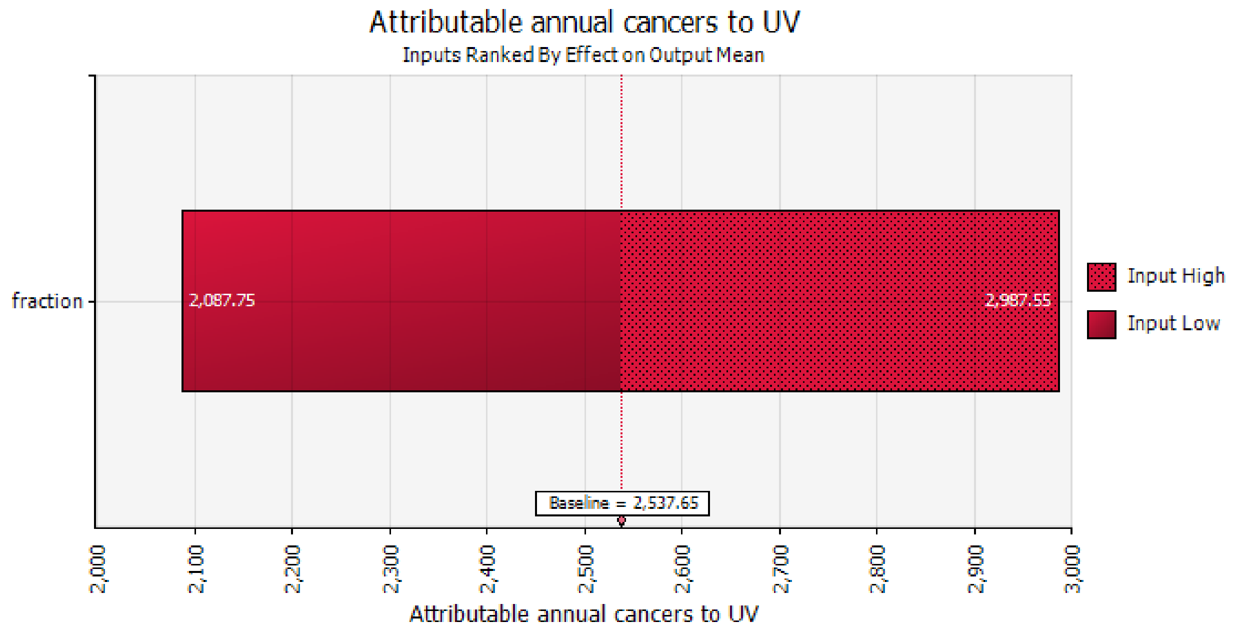

Figure 1. Tornado plot (indicating change in mean result if low/high value of distribution were used) for Ultraviolet Radiation

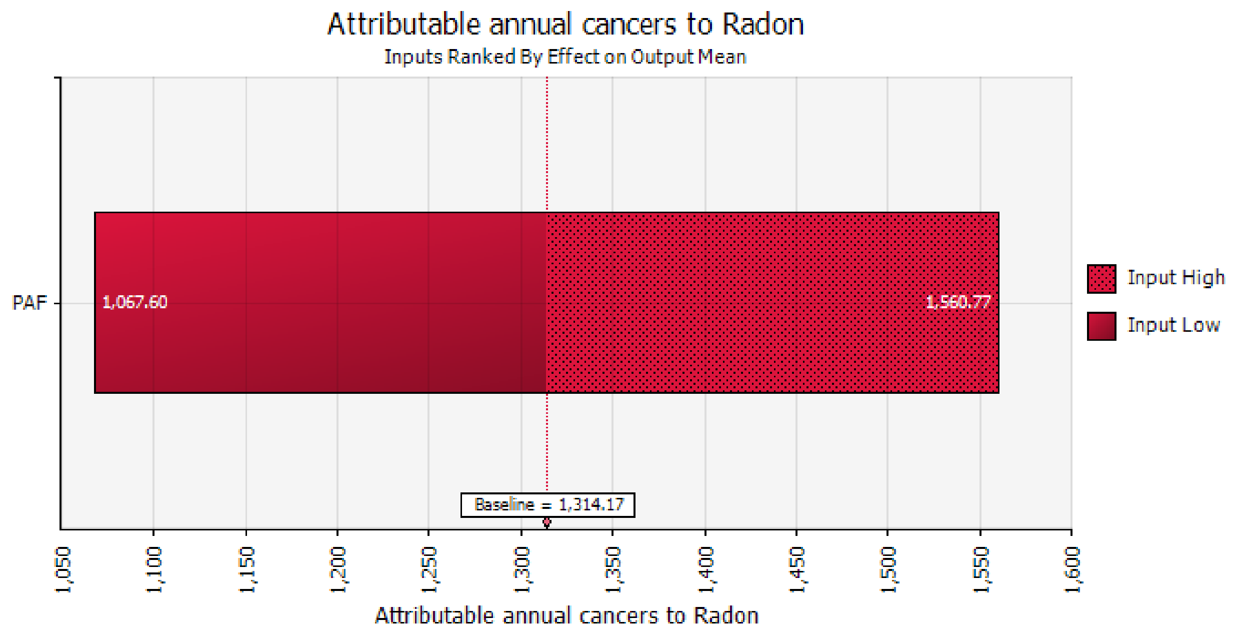

Figure 2. Tornado plot (indicating change in mean result if low/high value of distribution were used) for Radon

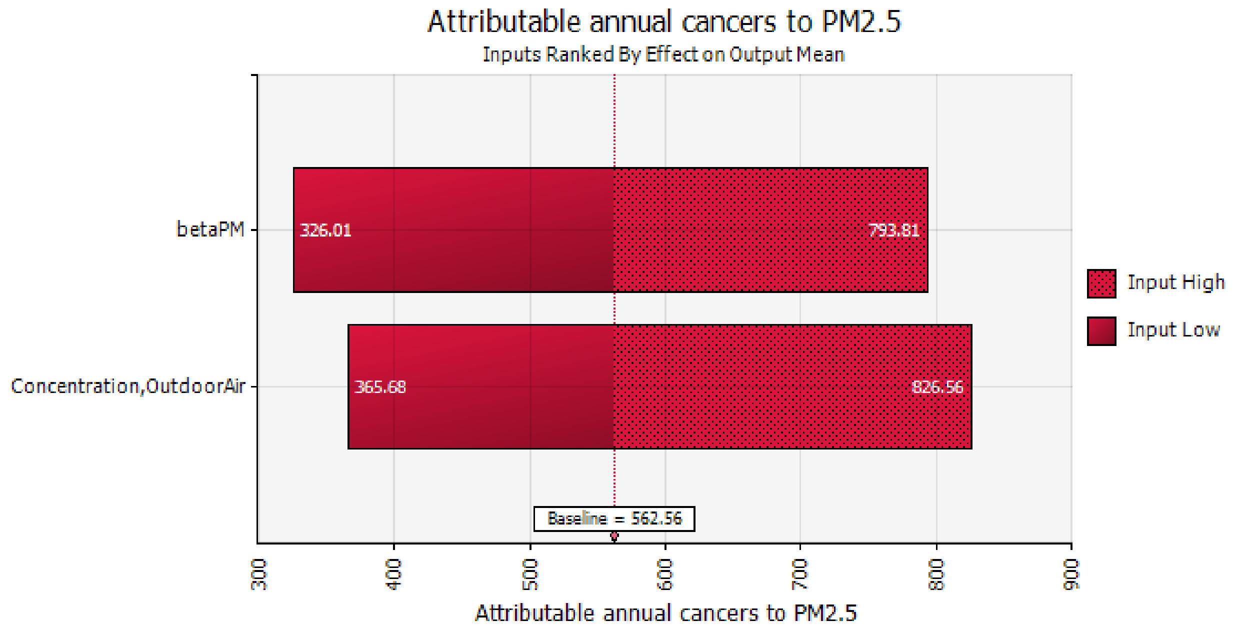

Figure 3. Tornado plot (indicating change in mean result if low/high value of distribution were used) for Fine Particulate Matter ($PM_{2.5}$)

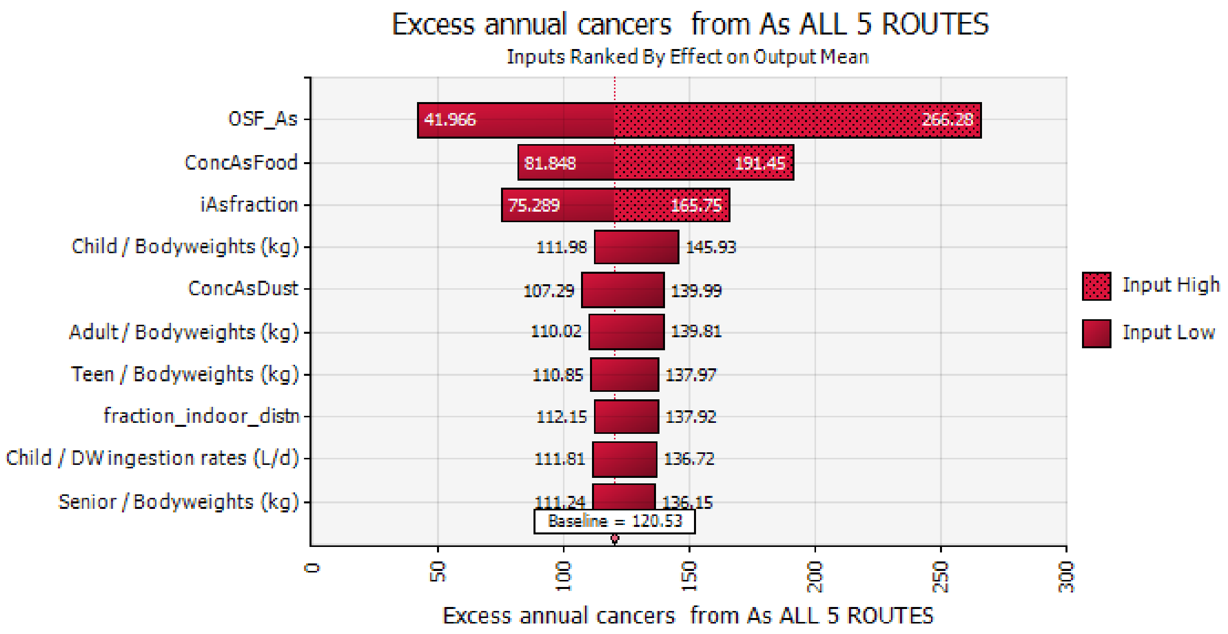

Figure 4. Tornado plot (indicating change in mean result if low/high value of distribution were used) for Arsenic

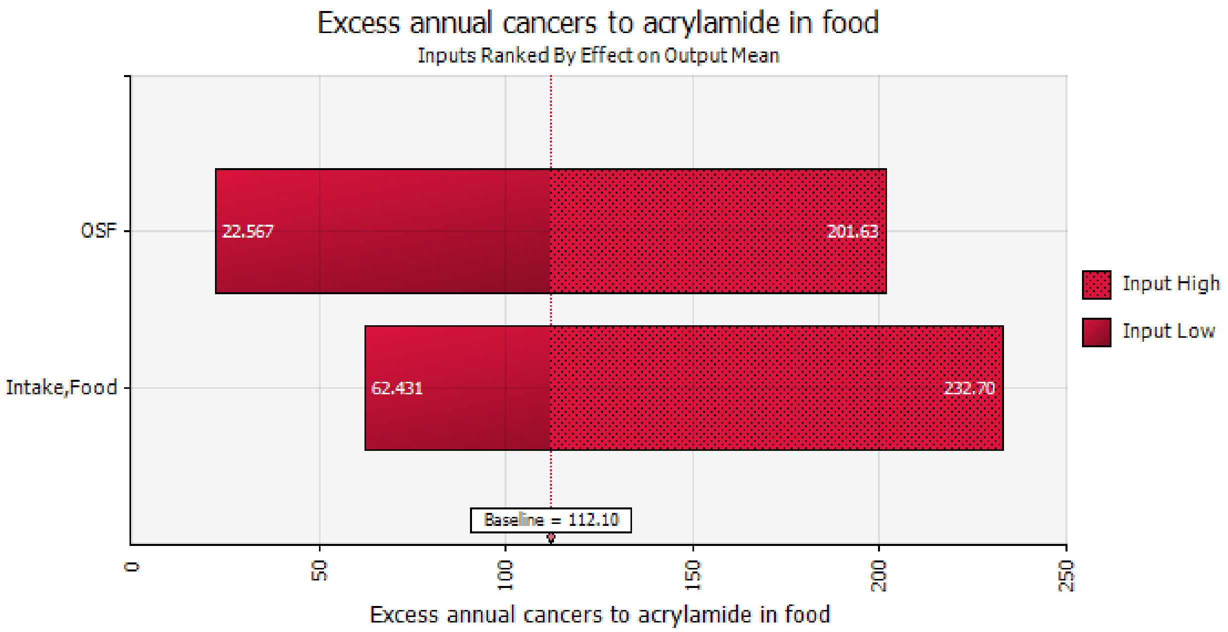

Figure 5. Tornado plot (indicating change in mean result if low/high value of distribution were used) for Acrylamide

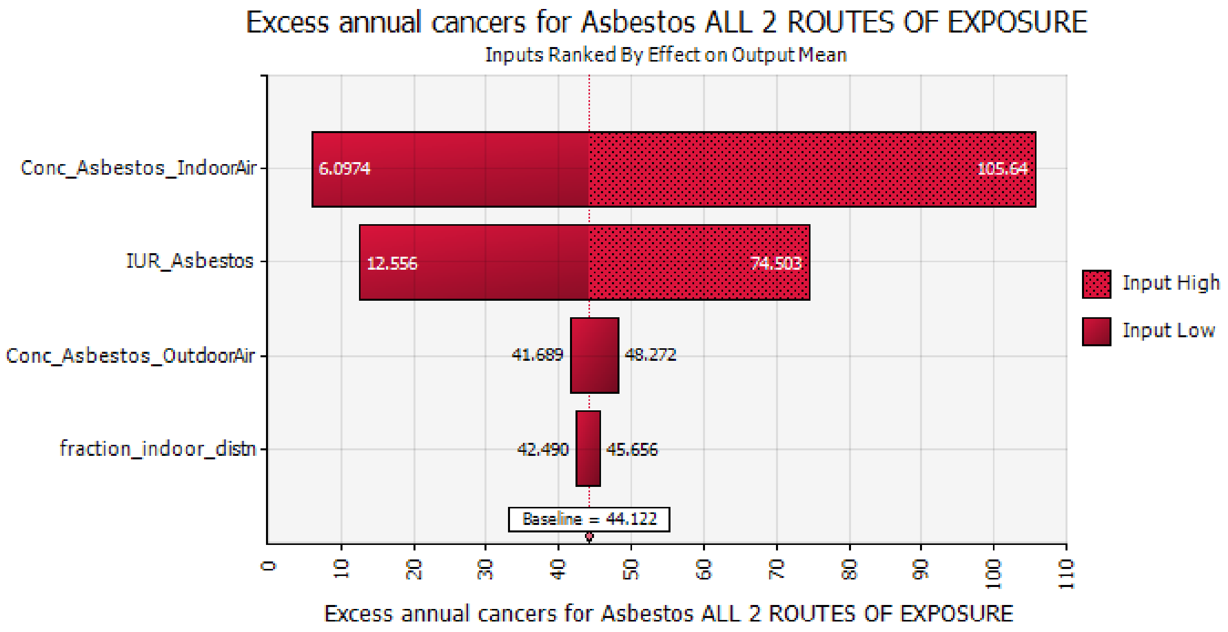

Figure 6. Tornado plot (indicating change in mean result if low/high value of distribution were used) for Asbestos

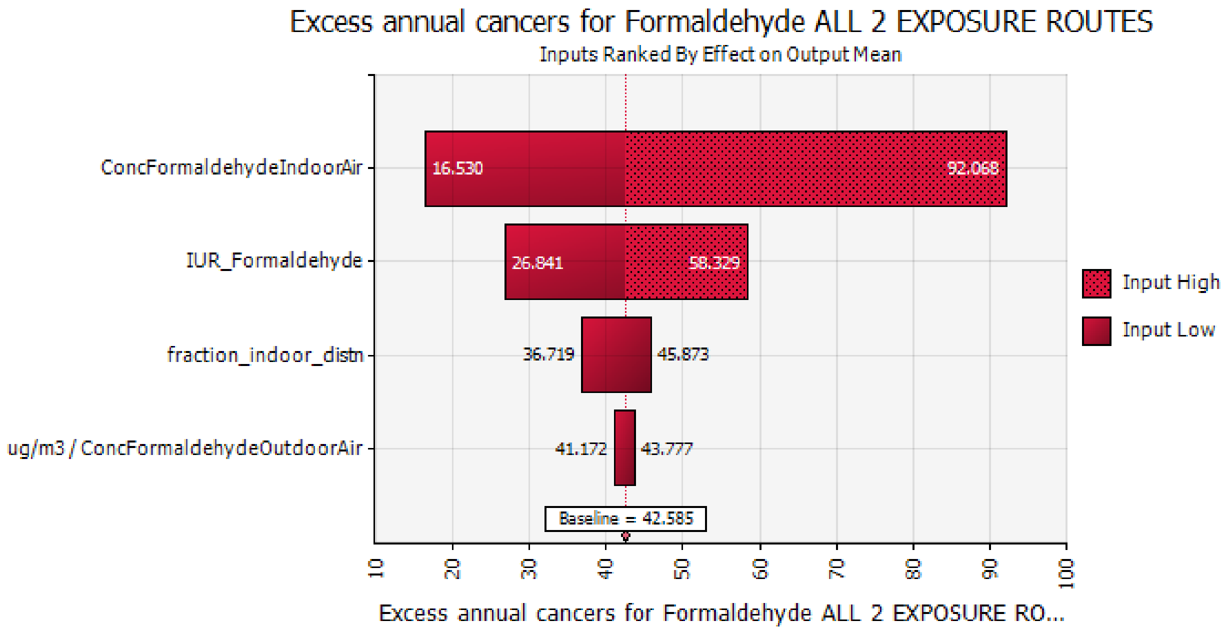

Figure 7. Tornado plot (indicating change in mean result if low/high value of distribution were used) for Formaldehyde

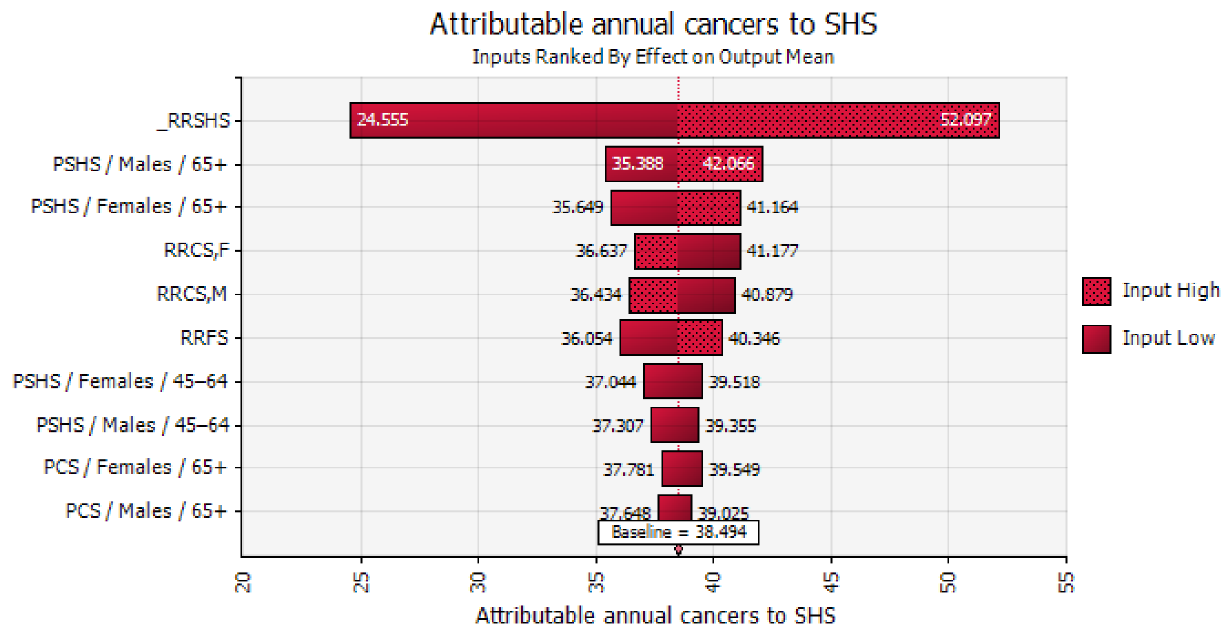

Figure 8. Tornado plot (indicating change in mean result if low/high value of distribution were used) for Secondhand Smoke

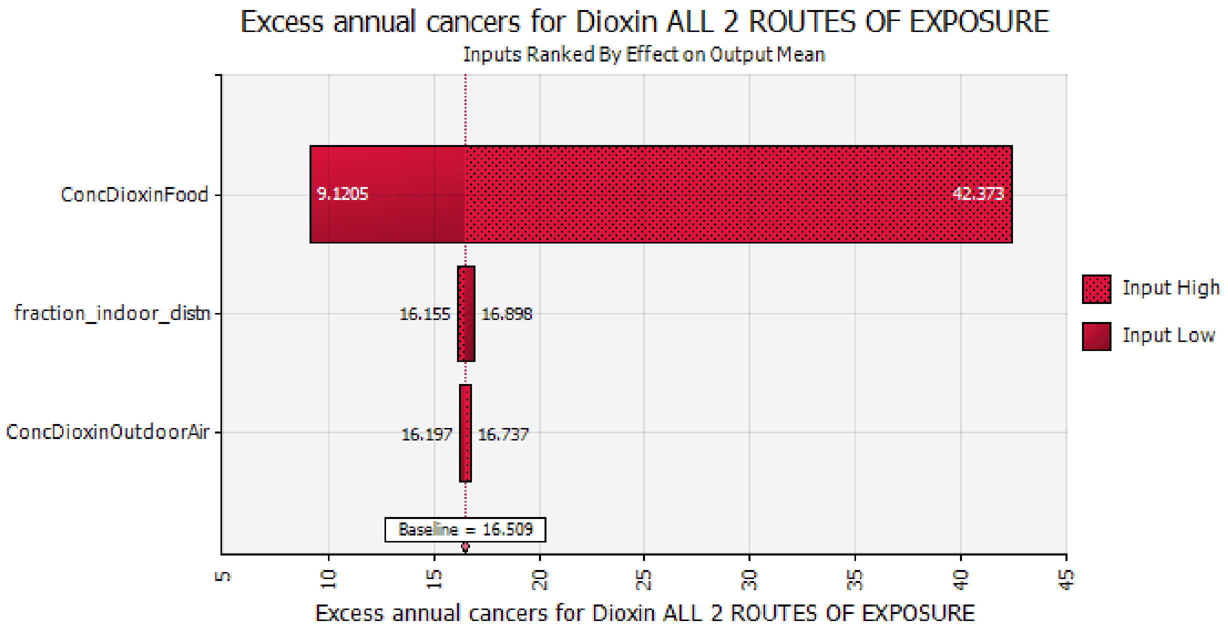

Figure 9. Tornado plot (indicating change in mean result if low/high value of distribution were used) for Dioxin

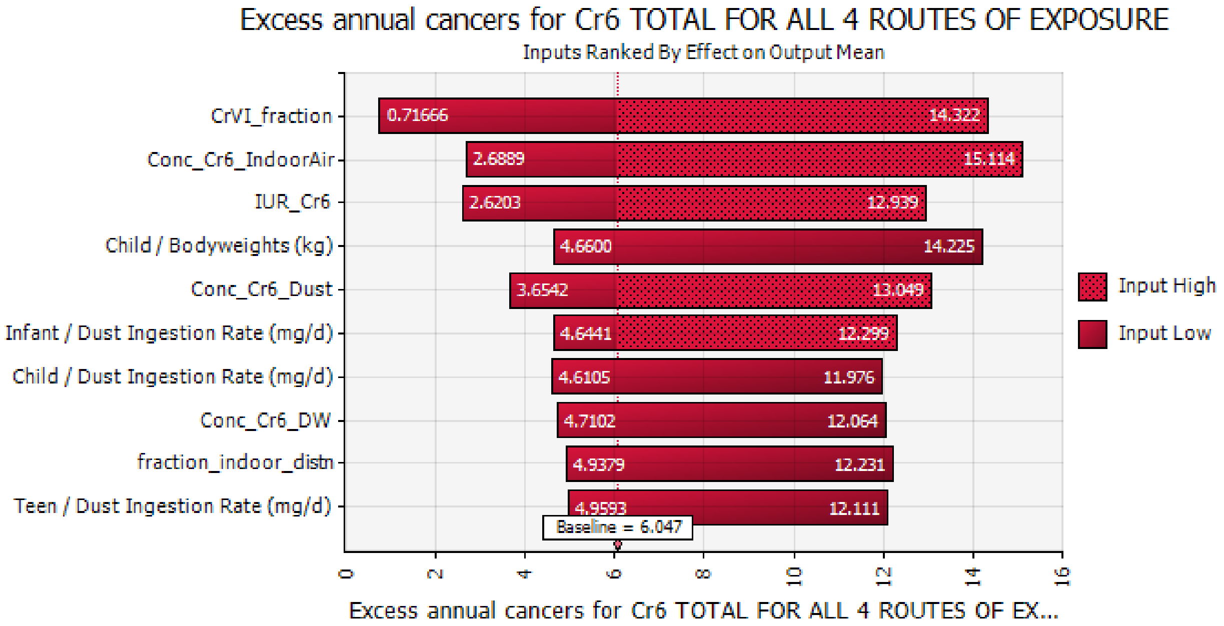

Figure 10. Tornado plot (indicating change in mean result if low/high value of distribution were used) for Chromium
